# miR-29a-5p Inhibits Prenatal Hair Placode Formation Through Targeting EDAR by ceRNA Regulatory Network

**DOI:** 10.3389/fcell.2022.902026

**Published:** 2022-05-12

**Authors:** Yao Jiang, Huatao Liu, Quan Zou, Shujuan Li, Xiangdong Ding

**Affiliations:** ^1^ National Engineering Laboratory for Animal Breeding, Laboratory of Animal Genetics, Breeding and Reproduction, Ministry of Agriculture, College of Animal Science and Technology, China Agricultural University, Beijing, China; ^2^ Anhui Provincial Key Laboratory of Livestock and Poultry Product Safety Engineering, Institute of Animal Husbandry and Veterinary Medicine, Anhui Academy of Agricultural Sciences, Hefei, China

**Keywords:** hair placode formation, ceRNA, miRNA-29a-5p, EDAR, cell proliferation

## Abstract

Hair placode formation is an important stage of hair follicle morphogenesis and it is a complex process facilitated by non-coding RNAs. In this study, we conducted whole transcriptome sequencing analysis of skin, heart, liver, lung, and kidney tissues of day 41 (E41) normal and hairless pig embryos, and respectively detected 15, 8, and 515 skin-specific differentially expressed (DE) lncRNAs, miRNAs, and mRNAs. Furthermore, 18 competing endogenous RNA (ceRNA) networks were constructed. Following weighted gene co-expression network analysis (WGCNA) of stages E39, E41, E45, E52, and E60, between normal and hairless pig embryos, only two ceRNAs (lncRNA2162.1/miR-29a-5p/BMPR1b and lncRNA627.1/miR-29a-5p/EDAR) that showed period-specific differential expression in E41 skin were retained. Dual-luciferase reporter assays further indicated that *EDAR* was a direct, functioning target of *miR-29a-5p* and that no binding site was found in BMPR1b. Moreover, *miR-29a-5p* overexpression inhibited the mRNA and protein expression of *EDAR* while no significant differential expression of BMPR1b was detected. In addition, over-expressed lncRNA627.1 reduces the expression of *miR-29a-5p* and increase EDAR expression while inhibits lncRNA627.1 resulted in a opposite expression trend. Cell proliferation result demonstrated that lower expression of EDAR and lncRNA627.1 inhibited hair placode precursor cells (HPPCs) proliferation in a manner similar to that shown by over-expressed *miR-29a-5p*. This study identified that *miR-29a-5p* inhibited HPPCs proliferation *via* the suppression of *EDAR* expression in the EDA/EDAR signaling pathway, while lncRNA627.1 rescues EDAR expression. Our study provides a basis for a better understanding of the mechanisms underlying the ceRNA complex, miR29a-5p/EDA*R/*lncRNA627.1, that could regulate hair placode formation, which may help decipher diseases affecting human hair.

## 1 Introduction

Hair follicle (HF) development is a complex morphogenetic process which occurs in two stages: prenatal hair morphogenesis development, and postnatal hair cycle development (Choi, 2018). Embryonic hair follicle morphogenesis development, which involves mesenchymal and epithelial interactions, begins with the formation of a hair placode in the early epidermis (Duverger and Morasso, 2009; Rishikaysh et al., 2014; Saxena et al., 2019). Therefore, placode formation is key to successful hair follicle morphogenesis. Morphological studies have indicated that hair placodes are formed on day 14.5 (E14.5) in mouse embryos, and during the 12th week [E55–E65 (∼E60)] in humans and Cashmere goats (Duverger and Morasso, 2014; Gao et al., 2016). Previous findings of ours have indicated that induction stage of pig hair follicle morphogenesis took place at E41 which the characteristic structure of this stage is hair placode formation (Jiang et al., 2019; Jiang et al., 2021). Thus, in pigs, E41 is the critical point which pig hair follicle morphogenesis begin to develop and hair placode start to form in this point and formation. Placode formation in developing embryonic skin requires stepwise signaling between the epithelial epidermis and the mesenchymal dermis. In recent years, many functional studies have uncovered the essential roles played by major signaling pathways, such as the Wnt/β-catenin, EDA/EDAR/NF-κB, and BMP pathways, in placode formation (Rishikaysh et al., 2014; Saxena et al., 2019). Moreover, numerous key genes that play a crucial role in hair placode formation, including *Wnt10b* (Andl et al., 2002), *CTNNB1* (Gay et al., 2015), *LHX2* (Tomann et al., 2016)*,* and *EDA/EDAR* (Zhang et al., 2009) which induce and maintain placode formation, as well as *BMP4* (St-Jacques et al., 1998) and *DKK4* (Fliniaux et al., 2008) which exert inhibitory effects on placode formation, have been identified.

Previous studies were primarily focused on gene function associated with the genetic regulation of placode formation. However, in gene regulation process, whether noncoding RNAs [long noncoding RNA (lncRNA) and microRNAs (miRNA)] are involved by influence gene function at various stages of HF morphogenesis and HF cycling development remains unclear (Andl and Botchkareva, 2015; Ge et al., 2019). Several specific miRNAs that play multiple roles in the regulation of HF development include *miR-22-5p* (Yan et al., 2019a) and *miR-203a-3p* (Luo et al., 2021) in hair follicle stem cells (HFSCs), *miRNA-203* (Ma et al., 2021) and *miR-199a-5p* (Han et al., 2020) in hair follicle development, as well as miR-195-5p in dermal papilla (Zhu et al., 2018), and miR-218-5p in hair shaft growth (Zhao et al., 2019), among others. Furthermore, biogenesis of canonical miRNAs depends on the cytoplasmic processing of pre-miRNAs to mature miRNAs by the Dicer endoribonuclease (Vishlaghi and Lisse, 2020) while Dicer deletion in epidermal result in hair follicle stem cell markers and degenerated (Andl et al., 2006), hair shaft depigmented (Liu et al., 2018a), failure of dermal papilla or hair-follicle maintenance (Teta et al., 2012), a decline in cell proliferation and increased apoptosis in hair follicle (Yi et al., 2006; Yi et al., 2009). In addition, lncRNAs, such as MSTRG.223165/miR-21/SOX6 in wool HF development (Zhao et al., 2020), as well as lncRNA5322/miR-19b-3p/MAPK1(Cai et al., 2019) and XR_310320.3/chi-miR-144-5p/HOXC8 (Ma et al., 2019) in hair follicle stem cells (HFSCs), also act as competitive endogenous RNAs (ceRNAs) to sponge miRNA and relieve the inhibitory effects exerted by these miRNAs on target genes during the regulation of HF development. Although the role of ncRNAs in HF development has been gradually deciphered, molecular mechanisms underlying the role played by ncRNAs, particularly the ceRNA regulatory networks, in the formation and maintenance of placodes remain unclear. Although a few studies have revealed that lncRNA2437 (Yue et al., 2016), miRNA195 (Liu et al., 2018b) and XLOC297809 (Nie et al., 2018) are involved in hair follicle initiation and placode induction, the genetic mechanisms underlying these processes have not yet been elucidated. A previous study of ours showed that hairless pigs were mainly caused by the blockage of hair placode formation at E41 in induction stage of pig hair follicle morphogenesis (Jiang et al., 2019). However, the precise molecular basis of blocked hair placode formation in pigs remains elusive, while the role of miRNA and lncRNA in hair placode formation remains unexplored as well.

Therefore, the objective of the current study was to screen out key candidate ceRNAs which may exert significant effects on hair placode formation *via* whole-transcriptome sequencing. Different tissues (skin, heart, liver, lung, and kidney) at E41 and different periods (E39, E41, E45, E52, and E60) in skin were sequenced to screened tissue- and periods-specific expression RNAs at E41 skin. Furthermore, the ceRNA based regulatory mechanism underlying hair placode formation was verified at the cellular level. Thus, our study of pig models should enhance future exploration of the hitherto unrecognized role played by the ceRNA network in hair placode formation in humans.

## 2 Materials and Methods

### 2.1 Ethics Approval

All animal procedures were evaluated and authorized by Institutional Animal Care and Use Committee (IACUC). The whole procedure for samples collected was carried out in strict accordance with the protocol approved by the IACUC at the China Agricultural University. The IACUC of the China Agricultural University specifically approved this study (permit number DK996).

### 2.2 Animals and Phenotype

E41 is a crucial stage for prenatal placode formation during hair follicle morphogenesis. In this study, tissues, including skin, heart, liver, lung, spleen, and kidney, from hairless (*n* = 3) and normal (*n* = 3) pig embryos at day 41 of gestation (E41). One embryo each from a litter, were sampled. In addition, mRNA sequencing of skin from hairless (*n* = 3) and normal (*n* = 3) embryos, one each from a litter, were also sampled at days 39 (E39), 45 (E45), 52 (E52), and 60 (E60) of gestation. The phenotype of every sample was identified following Hematoxylin and Eosin (HE) staining (Supplementary Figure S1). Hair follicle density was measured and those embryos with less than one hair follicle per cm^2^ and larger than four hair follicles per cm^2^ were determined as hairless and normal, respectively (Jiang et al., 2019).

### 2.3 RNAs (mRNA and ncRNA) Sequencing and Analysis

Total RNA was extracted using Trizol reagent (Invitrogen, Carlsbad, CA, United States), according to the manufacturer’s instructions. Quantity and purity were analyzed using a Bioanalyzer 2,100 and an RNA 6,000 Nano Labchip Kit (Agilent, Palo Alto, CA). Only RNA samples with suitable RNA electrophoresis results (28S/18S ≥ 1.0) and RNA integrity number (RIN) ≥ 7.5 could be analyzed further. The skin, heart, liver, lung and kidney tissues of normal (*n* = 3) and hairless (*n* = 3) embryos from the same litter were selected for mRNA, lncRNA and miRNA sequencing at E41. Embryo skins (normal = 3, hairless = 3) from the other four periods, namely E39, E45, E52, and E60, were selected for mRNA sequencing. mRNA and lncRNA were sequenced on Illumina HiSeq 4,000 and miRNA on HiSeq 2,000 platforms, which generated 150-bp paired-end and 50-bp single-end reads, respectively. Differentially expressed (DE) ncRNAs and mRNAs were identified using HISAT2 (Kim et al., 2019) and DESeq2 (Love et al., 2014) with an adjusted *p* value (false discovery rate, FDR) < 0.05 and a log_2_|FoldChange| > 1.

#### 2.3.1 mRNA Sequence Analysis

An Illumina high-throughput platform was used for mRNA sequencing and raw data was obtained using FastQC v0.11.9 (Chen et al., 2017). Quality control of the raw reads was conducted using NGSQCtoolkit_v2.3.3 (Patel and Jain, 2012). The *Sus scrofa* 11.1 reference genome (http://ftp.ensembl.org/pub/release-104/fasta/sus_scrofa/dna/Sus_scrofa.Sscrofa11.1.dna.toplevel.fa.gz) and gene sets file (http://ftp.ensembl.org/pub/release-104/gtf/sus_scrofa/Sus_scrofa.Sscrofa11.1.104.gtf.gz) were downloaded from Ensemble. HISAT2 (v2.0.4) and StringTie (v1.3.1) (Pertea et al., 2016) were performed to obtain clean reads aligned to the reference genome and mRNA reads were assembled for each sample. DESeq2 (Love et al., 2014) package was used for differential gene expression analysis.

#### 2.3.2 lncRNA-Seq Analysis

Quality control and mapping methods of lncRNA-seq data were similar to those used for mRNA-seq data. Candidate lncRNAs were selected using the following conditions: 1) Transcript length ≥200 and exon number ≥2, and Fragments Per Kilobase per Million reads (FPKM) >0.5; 2) Minimal read coverage ≥3 in at least one sample; 3) Filter known non-lncRNA annotations; and 4) Classify selected candidate lncRNAs, using the following four tools to predict coding potential: CPC2 (Kang et al., 2017), CNCI (Sun et al., 2013), PhyloCSF (v20121028) (Lin et al., 2011), and Pfam Scan databases (Finn et al., 2016). DESeq2 package was used to identify differentially expressed lncRNA between different groups at p_adj_ < 0.05. The lncRNA function depends on protein coding genes *via cis* and *trans* acting elements. Cis-acting (in which lncRNAs act on adjacent genes within a 100 kb distance) and trans-acting (in which the Pearson correlation of mutual expression levels is ≥0.95 or ≤−0.95) are widely adopted to forecast lncRNA gene interactive pairs. Bedtools (Quinlan and Hall, 2010) was used to identify neighboring genes approximately 100 kb upstream and downstream of differentially upregulated and downregulated lncRNAs, respectively.

#### 2.3.3 microRNA-Seq Analysis

Quality control methods used for miRNA-seq data were similar to those used for mRNA-seq data, where an NGSQCtoolkit_v2.3.3 was used. BWA software (Tam et al., 2015) was used for mapping and pig reference miRNA. Novel miRNA was predicted *via* miRDeep2 (Friedlander et al., 2012). The DESeq2 package was performed to identify miRNAs that were differentially expressed between different groups and a screening threshold for identifying differentially expressed miRNA was established at p_adj_ < 0.05. The target genes of miRNAs were predicted using Target Scan (http://www.targetscan.org/mamm_31/), RNA hybrid (https://bibiserv.cebitec.uni-bielefeld.de/rnahybrid/) and miRWalk v3.0 database (http://mirwalk.umm.uni-heidelberg.de/).Google charts (https://developers.google.com/chart/) were used to illustrate the targeting relationship between miRNA and mRNA. GO and Pathway enrichment analysis was performed using g:Profiler (https://biit.cs.ut.ee/gprofiler/gost) and ToppGene (https://toppgene.cchmc.org/enrichment.jsp), which terms with a adjusted *p* value (false discovery rate, FDR) greater than 0.05 were filtered.

### 2.4 Integrated ceRNA Regulatory Network

To investigate the role and interactions between ncRNAs and mRNAs during HF morphogenesis, ceRNA regulatory networks were constructed. The targeted relationships between lncRNA and miRNA were predicted *via* miRanda. Next, regulatory networks of lncRNA–miRNA–mRNA pairs were constructed based on co-location and co-expression (Tay et al., 2014). Two expression trend models, namely lncRNA downregulated |miRNA upregulated |mRNA downregulated (lncRNA−|miRNA+|mRNA−) and lncRNA upregulated |miRNA downregulated |mRNA upregulated (lncRNA+|miRNA−|mRNA+) were used to establish ceRNA regulatory networks. All networks were graphically displayed using Cytoscape3.6 (Shannon et al., 2003).

### 2.5 Weighted Gene Co-Expression Network Analysis

The mRNA sequencing of 30 embryo skins from five different stages (WGCNA_skins_periods; E39, E41, E45, E52, and E60) of normal and hairless pigs were integrated into the gene expression matrix for WGCNA. Then mRNA-seq data, which contained the expression data of 14,555 genes and 15,160 genes (Sum counts >10) for WGCNA_E41_tissues and WGCNA_skins_periods, was used to construct the gene expression matrix. Variance stabilizing transformation (VST) (Anders and Huber, 2010) function in the DESeq2 package was used to normalize and transform the two gene expression matrices respectively. The construction of two gene co-expression networks was based on the WGCNA package (Pei et al., 2017). Gene co-expression networks must conform to scale-free characteristics and obey power law distribution. Following sensitivity analysis of scale-free topology, the soft threshold power parameters of the two networks were set at 10 and 12, respectively (Ravasz et al., 2002).

### 2.6 Cell Culture

Approximately 2 cm^2^ skin samples from the lateral backsides of the pig embryo at days 41 gestation were surgically removed following standard, aseptic procedures. The tissues were cut at the interface of the sub cutaneous adipose layer and dermis. The remaining tissues were sub merged in 0.25% dispase II (Gibco, Grand Island, United States) overnight at 4°C to separate the epidermis from the dermis, following which the epidermis was cut into small blocks of approximately 1 mm^3^ in size, which were used as explants to culture hair placode precursor cells (HPPCs). The culture medium [DMEM (Gibco, Grand Island, United States) was supplemented with 10% FBS (Gibco, Grand Island, United States) and 1% penicillin-streptomycin (Gibco, Grand Island, United States)]. Normally, FBCs grew from each explant approximately 5 days after initial adhesion and were passaged when they reached 100% confluency. Typically, FBCs from the 3rd passage were used for all experiments. Human embryonic kidney (HEK)-293T (National Biomedical Cell Collection, Beijing, China) were cultured for Dual-Luciferase assay in DMEM supplemented with 10% FBS. All cells were incubated at 37°C in a 5% CO_2_.

### 2.7 Transfections

FBCs were seeded at 3 × 10^5^ cells per well in a 12-well plate and grown for 12 h. Then, the cells were transfected with 50 pmol of negative control of *ssc-miR-29a* mimics (mimics-NC, *n* = 3), *ssc-miR-29a* mimics (*n* = 3), 100 pmol of negative control of *ssc-miR-29a* inhibitor (inhibitor-NC, *n* = 3) or *ssc-miR-29a* inhibitor (*n* = 3), and 20 pmol of negative control EDAR siRNA (EDAR-NC, *n* = 3) or EDAR siRNA (*n* = 3), using Lipofectamine 3,000 (Invitrogen, Carlsbad, CA, United States) according to the manufacturer’s instructions. The mimics and inhibitor were designed and synthesized by RIBOBIO (Guangzhou, China). Total RNA and protein were isolated from the transfected cells 48/72 h post-transfection for further analysis.

### 2.8 Cell Proliferation Assay

Cell proliferation was assessed using a CCK-8 Cell Counting kit (Beyotime, Shanghai, China). FBCs were seeded in a 96-well plate (2 × 10^3^ cells per well) and then cultured for 12, 24, 36, 48, 60, or 72 h before adding 10 μl of CCK-8 (5 mg/ml) to the culture medium in each well. After incubating for 2 h, absorbance at 450 nm was measured using a Thermo-max microplate (ThermoFisher, Massachusetts, United States) reader. All experiments involving each transfection were performed in triplicate. HPPCs were transferred to culture medium with 50 µM 5-ethynyl-20-deoxyuridine (Edu, Beyotime, Beijing, China) for 2 h at 37°C after 36 h transfection. Afterwards, cells were fixed in 4% paraformaldehyde for 15 min at room temperature (RT), and then permeabilized with 0.3% Triton X-100 for 10 min. To block non-specific binding, cells were incubated in blocking buffer (PBS containing 3% bovine serum albumin, 0.3% Triton X-100) for 1 h at room temperature. Next, the cells were incubated with a solution containing 10 mM Edu for 30 min in the dark. Nuclei were stained with 10 μg/ml 4, 6-diamidino-2-phenylindole (DAPI, Solarbio, Beijing, China) solution in the dark for 10 min. A Leica SP8 confocal microscope was used to capture three randomly selected fields to visualize the number of Edu-stained cells.

### 2.9 Immunofluorescence Staining

Cells were fixed in 4% paraformaldehyde for 15 min after 48 h transfection and then permeabilized in 0.3% Triton X-100 for 10 min. Subsequently, cells were blocked with blocking solution (3% BSA, 0.3% Triton X-100, 10% FBS complemented with PBS) for 2 h. Then, immunofluorescence staining was performed using anti-EDAR (ABclonal, Guangzhou, China; 1:200) overnight at 4°C. Next, cells were stained with Alexa 594-labeled anti-rabbit antibody (Antgene, Wuhan, China; 1:200) for 1 h. Cell nuclei were visualized using DAPI (Solarbio, Beijing, China) solution in darkness for 10 min. Three randomly selected images were used to perform analysis.

### 2.10 Western Blotting

Western blotting (WB) was used to detect protein quantity. In brief, the sample was lysed in RIPA lysis solution, to extract total protein, and denatured. Next, the sample proteins were separated *via* SDS-PAGE gel electrophoresis under conditions involving a constant voltage of 100 V. In this study, the proteins were transferred from SDS-PAGE gel to PVDF membranes using a semi-dry transfer method. Then the membranes were blocked for 4 h, and incubated with primary antibodies against EDAR (ABclonal, 1:2000) and BMPR1b (ABclonal, 1:1500) overnight at 4°C. Next, the membranes were washed thrice with TBST (Trisbuffered saline) and incubated with secondary antibodies (1:1200 dilution) at room temperature for 1.5 h. Finally, a BeyoECL plus kit (Beyotime) was used to detect the protein signal. GAPDH (ABclonal, 1:1500) was used as a reference protein and blots were analyzed using IPWIN software.

### 2.11 Quantitative RT-PCR Analysis

Total RNA was isolated from dorsal pig skin with TriZol (Invitrogen, SanDiego, CA) following the manufacturer’s instructions. Next, cDNA was reverse transcribed using a PrimeScriptTM RT reagent kit with gDNA Eraser (Takara, Kyoto, Japan). RT-PCR was performed with LightCycler 480 SYBR Green I Master (Roche, Mannheim, Germany) mix on a LightCycler 480 real-time PCR system. GAPDH was used as a normalized control and relative gene expression was calculated based on the 2^−ΔΔCT^ formula. Measurements were recorded in triplicate. The following PCR conditions used: 95°C “hot start” for 10 min; 35 cycles of 95°C for 10 s, 60°C for 10 s, and 72°C for 10 s; and 72°C for 5 min. Primer sequences are provided (Supplementary Table S3). Differences between the gene expression of normal and hairless pigs were measured *via* a t test.

## 3 Results

A summary of the descriptive statistics of lncRNA-seq, miRNA-seq, and mRNA-seq data pertaining to different tissues, indicating the relatively high-quality of transcriptome data in this study is shown (Supplementary Table S1). Furthermore, mRNA and ncRNA (lncRNA and miRNA) sequencing of skin, heart, liver, lung, and kidney tissues of three normal and three hairless pig embryos at E41 stage were performed, and the number of differentially expressed (DE) mRNAs and ncRNAs are shown (Table 1). The DE RNAs (mRNAs, miRNAs, and lncRNAs) are illustrated in detail (Supplementary Figure S2) and sample-to-sample correlation in the five tissues was high (Supplementary Figure S2).

**TABLE 1:** The summarizes of DE mRNAs and ncRNAs in skin, heart, liver, lung and kidney of normal and hairless pig embryos at E41.

mRNA/ncRNA	Expression	Skin	Heart	Liver	Lung	Kidney
mRNA	Upregulated	372	96	298	288	231
	Downregulated	299	227	122	51	70
	Total	671	323	423	339	301
lncRNA	Upregulated	2	3	22	5	8
	Downregulated	13	9	6	19	12
	Total	15	12	28	24	20
miRNA	Upregulated	9	36	13	0	3
	Downregulated	0	18	0	1	0
	Total	9	54	13	1	3

### 3.1 Specific Differential Expression of mRNA and ncRNAs in Pig Embryos Skin at E41

**FIGURE 1:**
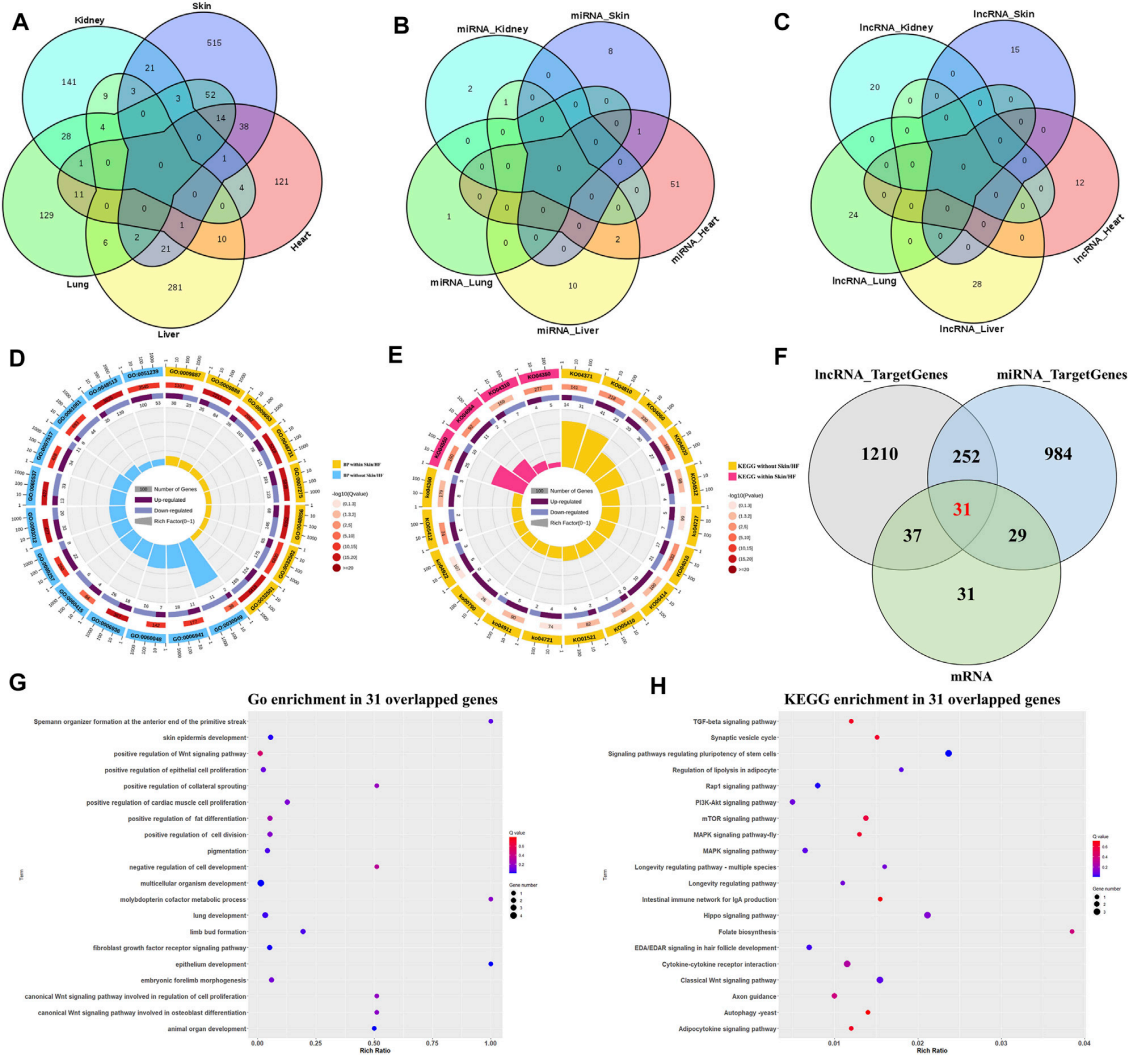
Differential expression of mRNAs, miRNAs, and lncRNAs between hairless and normal pig embryo skins at E41. Venn diagram for specific DE mRNAs **(A)**, DE miRNAs **(B)** and DE lncRNAs **(C)** in skin; **(D)** GO enrichment analysis for DE mRNAs; **(E)** KEGG enrichment analysis for DE mRNAs; **(F)** Venn diagram of candidate target genes of DE mRNA, DE miRNA and DE lncRNA at E41 skin; **(G)** Top 20 terms of GO enrichment in 31 overlapped target genes; **(H)** Top 20 terms of KEGG enrichment in 31 overlapped target genes.

Overlap between DE mRNAs, DE miRNAs and DE lncRNAs are illustrated (Figures 1A–C). In addition, 515 DE mRNAs (Figure 1A), 8 DE miRNAs (Figure 1B), and 15 DE lncRNAs (Figure 1C) were specifically differentially expressed between E41 skins of hairless and normal embryos. Next, qRT-PCR indicated that expression levels of randomly selected DE mRNA and ncRNA transcripts were highly consistent (Supplementary Figure S3), indicating the reliability of our RNA-seq data. Eight GO terms (Figure 1D) of the 515 DE mRNAs were significantly enriched in *skin development* and *epidermal development and differentiation*, while four hair follicle related signaling pathways, including the *Wnt* signaling pathway and the *BMP* signaling pathway*,* were identified (Figure 1E). Based on GO and pathway enrichment analysis, 128 of the 515 DE mRNAs (Supplementary Table S2) were selected which play important roles in HF development and hair placode formation. A total of 1,530 *cis* or *trans* target genes were predicted for the 15 DE lncRNAs in skin (Table 1), and among that, 68 target genes overlapped with those for the 128 DE mRNAs (Supplementary Figure S4A). Similarly, 1296 overlapped target genes were predicted for the 8 DE miRNAs by miRanda (3,452 target genes) and RNAhybrid (4,507 target genes); (Supplementary Figure S4B). Furthermore, *epithelium development, the Wnt signaling pathway*, the *EDA/EDAR signaling pathway*, and the *BMP signaling pathway*, among others, were enriched in these overlapped target genes (Supplementary Figures S3D,F), of which 60 genes overlapped with those of 128 DE mRNAs (Figure 1F; Supplementary Figure S4C). Thirty-one genes that overlapped between normal and hairless pig embryos at E41 stage skin tissue were identified based on DE mRNAs and target genes of DE miRNAs and lncRNAs (Figure 1F). These were enriched in hair follicle development, including skin *epidermis development* and *epithelial cell differentiation* (Figure 1G). Similarly, enriched KEGG pathways included the classical HF morphogenesis pathways such as the *Wnt*, *TGF-β,* and *EDA/EDAR signaling pathways* (Figure 1H). As well, the key genes *EDAR*, *DKK4*, *BMPR1B*, *BMP3*, *WNT3,* and *FGFR2,* among others, were identified.

### 3.2 Construction and Weighted Gene Co-Expression Network Analysis of ceRNA Regulatory Networks

**FIGURE 2:**
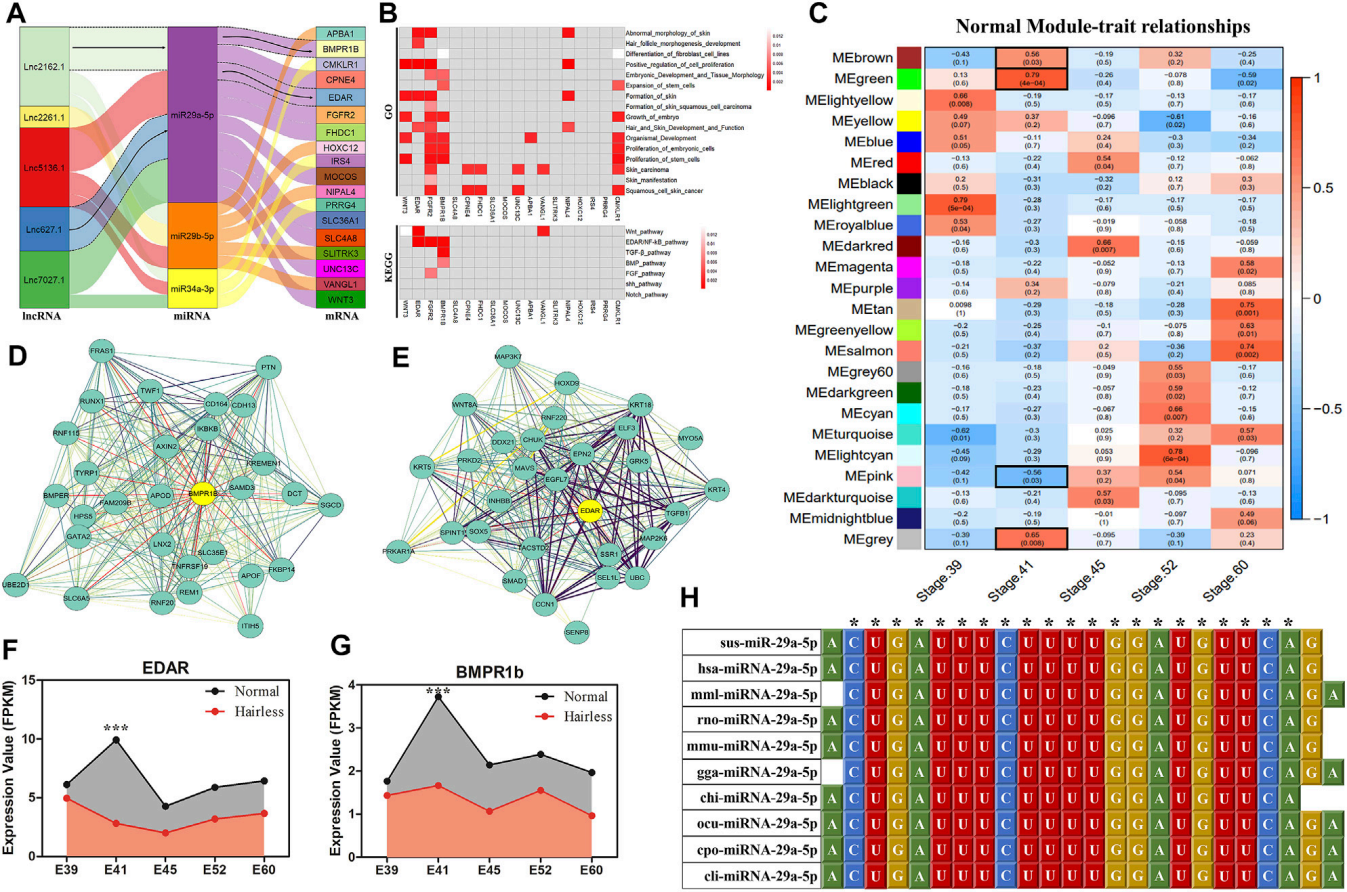
ceRNA Regulatory Networks Construction and WGCNA for hairless and normal pig skin during different periods. **(A)** Sankey diagram of the ceRNAs network in skin; Each rectangle represents a gene, and the degree of connection of each gene is expressed based on the size of the rectangle; **(B)** GO and KEGG enrichment analysis of the 18 DE target genes in ceRNAs; **(C)** Module-trait associations of normal pig skin during different periods; Each row corresponds to a module epigene, while each column corresponds to a period. Each cell contains the corresponding correlation and *p*-value; The table is color-coded by correlation according to the color legend; The black solid box represents the significance of the modules associated at E41; The interaction network of EDAR **(D)** and BMPR1b **(E)** in the MEgreen module of the normal group; The hub genes of EDAR and BMPR1B in the modules are denoted by yellow circles containing bolded text; the expression of EDAR **(F)** and BMPR1b **(G)** in skin during different periods (E39, E41, E45, E52, and E60) between normal and hairless pigs; **(H)** Sequence alignment and phylogenetic analysis of miRNA-29a-5p. Sus, *Sus scrofa*; hsa, *Homo sapiens*; mml, *Macaca mulatta*; rno, *Rattus norvegicus*; mmu, *Mus musculus*; gga, *Gallus gallus*; chi, *Capra hircus*; ocu, *Oryctolagus cuniculus*; cpo, *Cavia porcellus*; cli, *Columba livia*.

Based on expression trend models (lncRNA−|miRNA+|mRNA−) pertaining to the expression trends of 31 overlapping target genes as well as the lncRNAs and miRNAs regulating the expression of these genes, 18 ceRNA regulatory networks were constructed. These networks contained 18 overlapped DE genes, five DE lncRNAs, and three DE miRNAs (Figure 2A). Function and pathway enrichment analysis of GO (Figure 2B) showed that *EDAR*, *FGFR2*, *BMPR1b*, *and VANGL1* were enriched in classical hair follicle pathways, such as *Wnt*, *EDAR/NF-*κ*B*, *TGF-β, BMP*, and *FGF signaling pathway*s, and that the GO terms were mainly related to *skin morphology and formation*, *hair and skin development function* and *fibroblast cell line differentiation.*

To further screen ceRNAs for skin-specific expression at E41, WGCNA was performed on normal and hairless pig skin at five stages, namely E39, E41, E45, E52, and E60, using mRNA sequencing. The independence degree approximated 0.8 while the average connectivity degree was higher (Supplementary Figures S4A,C). In total, 24 and 28 distinct gene co-expression modules were constructed for the normal group and the hairless group, respectively (Supplementary Figures S4B,D). Modules showing *p* < 0.05 at E41 were selected, and interestingly, four modules, *MEbrown* (*p* = 0.03)*, MEgreen* (*P* = 4E-04)*, MEpink* (*p* = 0.03) and *MEgray* (*p* = 0.0083), were identified in normal pigs (Figure 2C), while no significant module was found in hairless pigs (Supplementary Figure S6). Among the hub genes of the four significant modules, only *EDAR* (Figure 2D) and *BMPR1b* (Figure 2E), of the 18 DE target genes in ceRNA, were found to be involved. Additionally, both *EDAR* and *BMPR1b* were interacted with many hub genes (such as *KRT5*, *WNT8A*, and *KRT4*) which were related to hair follicle development, in the most significant *MEgreen* module (Figure 2D). Moreover, the expression levels of EDAR (Figures 1A, 2F) and BMPR1b (Figures 1A, 2G) in skin at different periods, were found to be not only tissue-specific but also period-specific at E41 (*p* < 0.001). Therefore, two ceRNAs, lncRNA2162.1/miR-29a-5p/BMPR1b and lncRNA627.1/miR-29a-5p/EDAR, were retained. The results of *ssc-miR-29a-5p* sequence alignment in different species revealed that *ssc-miR-29a-5p* is highly conserved among species (Figure 2H), suggesting the importance of the role played by *ssc-miR-29a-5p*. A previous study of ours indicated that E41 constituted the critical stage of hair placode formation, and the results of our current study indicated that *EDAR* and *BMPR1b* may play an important role in hair placode formation *via* the ceRNA network.

### 3.3 *EDAR* Was a Target Gene of *miR-29a-5p* in ceRNA

**FIGURE 3:**
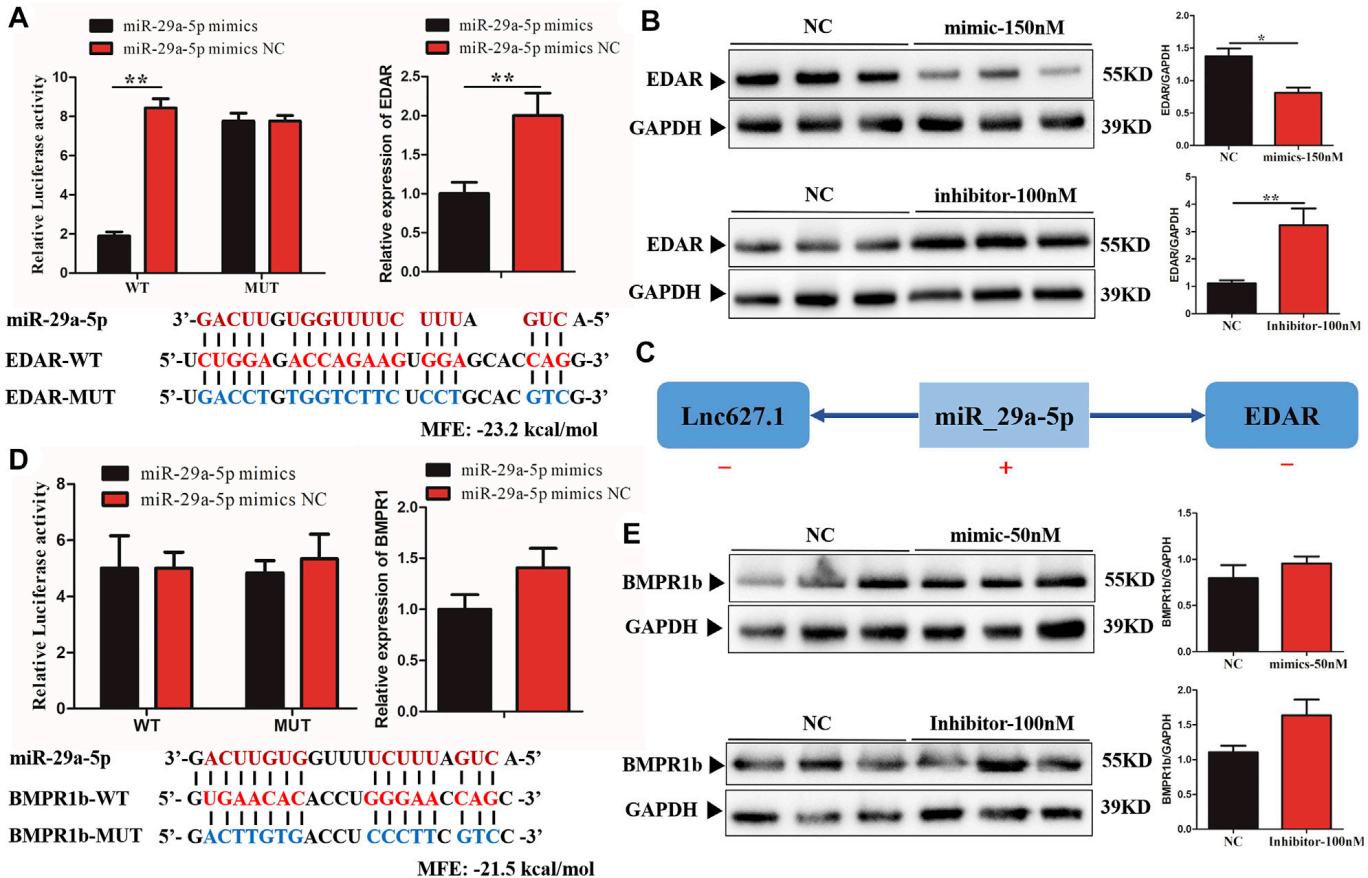
Verification of miR-29a-5p target binding for EDAR and BMPR1b 3′ UTR. **(A)** Left histogram: luciferase assays were performed in 293-Tcells co-transfected with pMirGLO-EDAR–5′-UTR-WT/MUT and ssc-miRNA-29a-5p mimics; Right histogram: EDAR mRNA expression detected in fibroblast cells after treatment with ssc-miRNA-29a-5p mimics; **(B)** EDAR protein expression detected in fibroblast cells following miRNA-29a-5p overexpression or inhibition. **(C)** The mechanism of ceRNA network expression in lncRNA627.1/miRNA-29a-5p/EDAR. **(D)** Left histogram: luciferase assays were performed in 293-Tcells co-transfected with pMirGLO-BMPR1b–3′-UTR-WT/MUT and ssc-miRNA-29a-5p mimics; Right histogram: BMPR1b mRNA expression detected in fibroblast cells after treatment with ssc-miRNA-29a-5p mimics; **(E)** BMPR1b protein expression detected in fibroblast cells following miRNA-29a-5p overexpression and inhibition. Red letters indicate wild type sites and blue letters indicate mutated sites in the pMir-report luciferase reporter vector. Error bars indicate the mean ± SD of triplicate experiments. *p < 0.05, **p < 0.01, ***p < 0.001. MFE, minimum free energy.

Among the ceRNAs, only *miR-29a-5p* was able to regulate *EDAR* and *BMPR1b*. In order to validate direct regulation of the expression levels of *EDAR* and *BMPR1b* by *miR-29a-5p*, a construct containing the 5′UTR of the potential target genes, or the sequence with the mutant seed region, was co-transfected together with *miR-29a-5p* mimics in human 293T cells. As predicted, *miR-29a-5p* mimics significantly reduced EDAR-WT-5′UTR (*p* < 0.01) luciferase activity, whereas no significant inhibition of EDAR-MUT-5′UTR was detected (Figure 3A). Furthermore, the mRNA and protein expression levels of *EDAR* were significantly decreased (*p* < 0.05) by *miR-29a-5p* mimics, whereas these levels were significantly increased (*p* < 0.01) by a *miR-29a-5p* inhibitor, compared with the effect exerted by *miR-29a-5p* on *EDAR* in the control group (NC) (Figures 3A,B). However, in regard to *BMPR1b*, no luciferase activity was detected in either BMPR1b WT or MUT-5′UTR, suggesting that *miR-29a-5p* was unable to bind BMPR1b 5′UTR (Figure 3D). In addition, no significant change was detected in the mRNA (Figure 3D) and protein (Figure 3E) expression of BMPR1b treated with *miR-29a-5p* mimics and an inhibitor, suggesting the absence of a binding site between *miR-29a-5p* and BMPR1b. Our results confirmed that *EDAR* is a direct and functionally relevant target of *miR-29a-5p*, the expression trend of which is illustrated (Figure 3C) and that both are involved in hair placode formation, possibly *via* ceRNA activity.

### 3.4 *miRNA-29a-5p* Inhibits Hair Placode Formation Through Targeting EDAR *via* the EDA/EDAR Signaling Pathway

**FIGURE 4:**
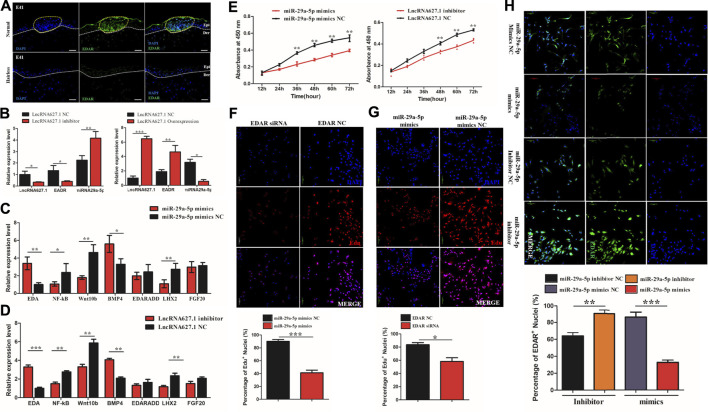
miRNA29a-5p regulates targeting of EDAR by the Wnt signaling pathway to inhibit hair placode formation. **(A)** Immunofluorescence (IF) of EDAR in the hair placode during hair follicle morphogenesis in prenatal normal and hairless embryos. **(B)** The mRNA expression of EDAR and miR-29a-5p under lncRNA627.1 overexpression and the presence of an inhibitor. **(C)** The mRNA expression of related genes involved in the EDA/EDAR signaling pathway, following miR-29a-5p overexpression. **(D)** The mRNA expression of related genes involved in the EDA/EDAR signaling pathway following transfection with a lncRNA627.1 inhibitor. **(E)** Proliferation of fibroblasts at 0, 24, 36, 48, 60, and 72 h after transfection of miR-29a-5p mimics and a lncRNA627.1 inhibitor, as well as miR-29a-5p mimics NC and lncRNA627.1 inhibitor NC. **(F)** Proliferating fibroblasts labeled with Edu after transfecting EDAR siRNA; **(G)** Proliferating fibroblasts labeled with Edu following transfection with miR-29a-5p mimics. The click-iT reaction revealed Edu staining (red). Cell nuclei were stained with DAPI (blue). **(H)** Protein expression of EDAR in fibroblasts following transfection of miR-29a-5p mimics, a miR-29a-5p inhibitor, miR-29a-5p mimics NC and lncRNA627.1 inhibitor NC, cell nuclei were stained with DAPI (blue) while EDAR proteins were stained green. **p < 0.05, **p < 0.01, ***p < 0.001.

To further verify the function of *EDAR* in hair placode formation, protein expression of EDAR in E41 skin of normal and hairless embryos was determined. *EDAR* was highly expressed in the hair placodes of normal embryos which consistent with previously reported (Schmidt-Ullrich et al., 2006; Zhang et al., 2009; Wu et al., 2020) while weakly expression in hairless embryos skin, suggesting that *EDAR* plays a key role in hair placode formation at E41 (Figure 4A). The competitive mechanism involving lncRNA627.1-miR-29a-5p-EDAR is illustrated (Figure 4B), lncRNA627.1 mimics significantly upregulated (*p* < 0.05) *EDAR* expression while downregulating *miR-29a-5p* expression (*p* < 0.01). Meanwhile, the lncRNA627.1 inhibitor group displayed a converse expression trend, where *EDAR* was downregulated and *miR-29a-5p* was upregulated (Figure 4B). To gain further insights into how lncRNA627.1 and *miR-29a-5p* regulate *EDAR*, related genes, *EDA*, *NF-ĸB*, *Wnt10b*, *BMP4*, *EDARADD*, *LHX2,* and *FGF20,* associated with the EDA/EDAR signaling pathway, were also detected after transfecting HPPCs with *miR-29a-5p* mimics and a lncRNA627.1 inhibitor. Among the *miR-29a-5p* mimics group, *BMP4* (*p* < 0.05) was remarkably upregulated while *EDA* (*p* < 0.01), *NF-κB* (*p* < 0.01), *Wnt10b* (*p* < 0.01) and *LHX2* (*p* < 0.01) were significantly downregulated (Figure 4C). Similar results were also observed in the lncRNA627.1 inhibitor group (Figure 4D). Our results demonstrated that the similar expression trend in EDAR related genes by lncRNA627.1 expression inhibition and *miR-29a-5p* overexpression which suggested that a regulation might existed between lncRNA627.1 and miR-29a-5p and need furthermore investigation to explore whether regulated by ceRNA (Figures 4C,D). In addition, proliferation of HPPCs further confirmed the regulation by *miR-29a-5p* and lncRNA627.1. Thirty-six h following the transfection of *miR-29a-5p* mimics and lncRNA627.1 inhibitor, the number of viable cells in the *miR-29a-5p* mimics group was significantly decreased (*p* < 0.01), and continued to decrease further for 48, 60, and 72 h, a result consistent with findings for the lncRNA627.1 inhibitor group (Figure 4E). We further explored the role played by the association between *EDAR* and *miR-29a-5p* in HPPCs proliferation, *via* siRNA-mediated EDAR silencing as well as *via miR-29a-5p* mimics mediated suppression of EDAR expression. Edu staining assays indicated that following the reduction of EDAR expression, the proportion of Edu^+^ HPPCs cells were reduced when transfected with EDAR siRNA (*p* < 0.001) (Figure 4F) or *miR-29a-5p* mimics (*p* < 0.05) (Figure 4G). Meanwhile, compared with that of mimics/inhibitor NC groups, *EDAR* ^*+*^ HPPCs cells were upregulated following transfection *miR-29a-5p* inhibitor (*p* < 0.01), while it was downregulated in the *miR-29a-5p* mimics group (*p* < 0.001) (Figure 4H). Considered together, our results indicated that *miRNA29a-5p* inhibits HPPCs proliferation by suppressing the expression of the targeting gene, *EDAR,* in the EDA/EDAR signaling pathway, while lncRNA627.1 removes such inhibition.

## 4 Discussion

Previous studies of ours (Jiang et al., 2019) indicated that impairment of hair placode formation at E41 constitutes the main cause of hairlessness in hairless pigs. In this study, whole transcriptome sequencing (mRNA, lncRNA, and miRNA) of normal and hairless pig embryos at E41 screened out *EDAR* and an associated regulatory mechanism involving ceRNAs. Of the known key genes involved in hair placode formation, *EDAR* appears to play an important, early role by regulating signaling molecules involved in the establishment, formation, and regulation of placodes (Saxena et al., 2019). High expression levels of *EDAR* increased the number and size of hair placodes (Mustonen et al., 2004; Mohri et al., 2008) whereas hair placode formation could neither be induced nor maintained in the absence of *EDAR* (Schmidt-Ullrich et al., 2006; Fliniaux et al., 2008). Impaired hair placode formation was associated with a reduction in the epidermal expression levels of *EDAR, Lef1, and Shh*, which are essential for hair follicle morphogenesis (Mohri et al., 2008).

In our study, EDAR was highly expression at the hair placodes of normal skin while no expression in hairless (Figure 4A) which not only confirmed the key role of EDAR in hair placode formation, but also showed consistency with the results of previous studies (Schmidt-Ullrich et al., 2006; Zhang et al., 2009; Wu et al., 2020). Furthermore, this study also demonstrated that high *EDAR* expression promotes the proliferation of HPPCs, while low *EDAR* expression inhibits such proliferation (Figure 4F). Previous studies had also reported that EDAR exerts its effect on epithelial (Jaskoll et al., 2003) and matrix progenitor cell proliferation (Wang et al., 2017a) *via* the EDA/EDAR/NF-κB signaling pathway. In hair follicles, Gata6 regulates the rapid proliferation of epithelial (matrix) progenitors during adult mouse hair follicle regeneration, by stimulating the activation of EDAR and NF-κB pathways (Wang et al., 2017a). Furthermore, EDAR and NF-κB play a role in keratinocyte proliferation and hair placode down-growth (Schmidt-Ullrich et al., 2006).

Besides that, it was necessary to use HPPCs in this study, due to the absence of a specific cell line that could be utilized for hair placode formation research. Although, it is advantageous to use tissue block culture and organoid culture methods to detect hair follicle related phenotype at the cellular level while still exist many problems. In tissue block culture for hair placode, the culture environment and culture additive such as activator, enzymes, small molecules, growth factors are still unknown in pig [Zhou et al. (2018), Yan et al. (2019b)] while the combination selection of multiple cell lines and precises space culture environment limit the development of organoid culture (Lee et al., 2018; Lee et al., 2020; Lee and Koehler, 2021). HPPCs at the E41 stage were used in this study because another research of us (unpublished) found that the formation of hair placode mainly comes from a kind of hair placode precursor cell (HPPCs) in epidermal (Supplementary Figure S7A), which not only specifically expresses EDAR, but also highly expresses fibroblast cells related marker genes (*COL1A1*, *LUM*, *Vimentin*), just similar to a pseudo fibroblast cell lineage (Supplementary Figure S7B). We speculated that this pseudo fibroblasts (HPPCs) might be the key cells leading to the formation of hair placode which could also screened from epidermal tissue and highly expressed FB marker, *Vimentin* and *LUM* (Supplementary Figure S7C). Thus, these results were the main basis for us to select HPPCs to research in this study. In addition, according to Chen *et al.*, fibroblast proliferation plays a functional role in hair placode initiation (Chen et al., 2012) while proliferation capacity was the determining factor in the initiation of placode formation (Duverger and Morasso, 2009; Schneider et al., 2009; Rishikaysh et al., 2014). Our study provided a speculation which EDAR could directly promote hair placode development *via* a pseudo fibroblasts lineage, HPPCs cells proliferation.

**FIGURE 5:**
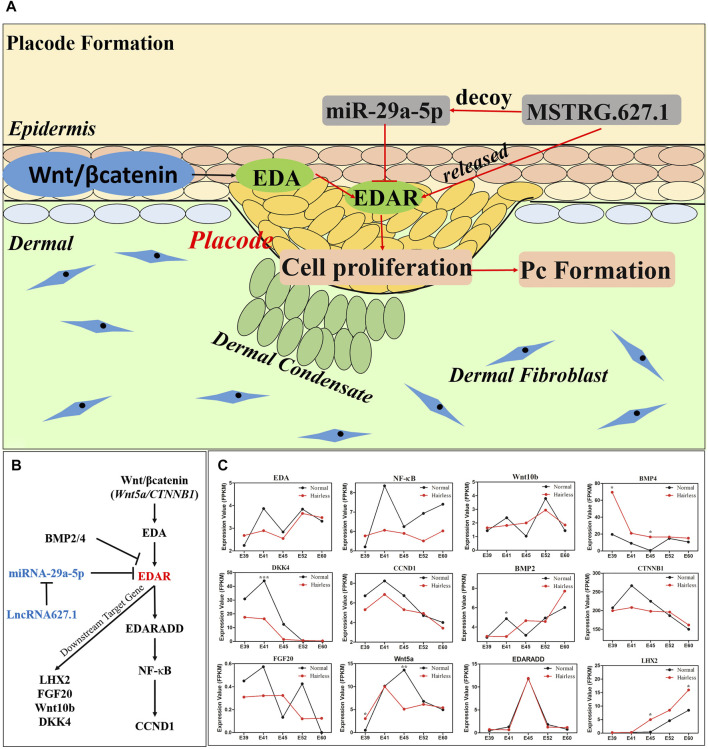
The molecular mechanism of lncRNA627.1/miR-29a-5p/EDAR involved in hair placode formation and expression of EDAR related genes detected in the EDA/EDAR signaling pathway. **(A)** miR-29a-5p inhibited cell proliferation to suppress the formation of hair placodes by targeting EDAR; **(B)** Schematic illustrating the association between downstream/upstream target gene expression and EDAR in the EDA/EDAR signaling pathway; **(C)** The expression of EDAR related genes, Wnt5a, CTNNB1, EDA, EDARADD, NFKB1, CCND1, FGF20, Wnt10b, DKK4, BMP2, and BMP4, in normal and hairless pig skins, at stages E39, E41, E45, E52, and E60.

Increasingly, evidence indicates that ncRNA participates in the regulation of hair follicle morphogenesis and development *via* the ceRNA network which imposes mutual regulation among miRNAs, lncRNAs, and mRNAs (Cai et al., 2019; Zhao et al., 2020). According to ceRNA theory, miRNA suppresses gene expression by recognizing specific target mRNAs, while lncRNAs, which act as natural miRNA sponges, inhibit miRNA function and modulate the expression of target mRNAs by interacting with miRNA response elements (Salmena et al., 2011). In our study, significantly high expression levels of *miR-29a-5p* suppressed the expression of *EDAR* and lncRNA627.1 in hairless pig skin tissue at E41 (Figure 2B). Meanwhile, overexpression lncRNA627.1 elevated the expression of *EDAR* while the expression of *miR-29a-5p* was reduce which suggested that lncRNA627.1 may disrupt the binding between miRNA and mRNA while competitive binding displacement assay was needed in further investigation (Figure 4B). Another similar regulatory mechanism based on ceRNAs is that of *miR-144-5p,* which indirectly affects HFSCs by targeting lncRNA310320.3 and lncRNA311077.2 (Ma et al., 2019). lncRNA679 promoted Wnt3 expression by targeting *miR-221-5p* in hair follicle cycle development (Wang et al., 2017b). Generally, ncRNA function involves regulation of the expression of targeted mRNAs (Salmena et al., 2011; Tay et al., 2014); for example, lncRNA5322 serves as a ceRNA of *miR-19b-3p* to elevate MAPK1 expression, ultimately promoting proliferation of HFSCs and epidermal regeneration in mouse models (Cai et al., 2019). Previous studies have reported that miR-29a plays a positive role in skin fibroblast protection (Zhou et al., 2016), hair follicle regeneration (Zhu et al., 2020), and HFSC proliferation (Guo et al., 2018; Ge et al., 2019) by regulating target genes. The results of the current study further revealed that *miR-29a-5p* plays a role in placode formation by regulating *EDAR*, where *miR-29a-5p* downregulated *EDAR* expression, thereby inhibiting HPPCs proliferation and hair placode formation. In contrast, lncRNA627.1 upregulated EDAR expression by suppressing *miR-29a-5p* expression (Figure 5A). It’s worth noting that current data supported a model where ncRNA627.1 acts upstream of miR-29a expression (Figures 4B–E), but no direct evidence that lncRNA627.1 could release the EDAR expression by competitively binding *miR-29a-5p* based on ceRNA regulatory mechanism. Thus, the relation between lncRNA627.1 and miR-29a-5p, such as competitive binding displacement assay, needs to be further verification.

According to previous studies, a complex regulatory mechanism involving multiple genes and pathways underlies hair placode formation (Duverger and Morasso, 2009; Schneider et al., 2009; Sennett and Rendl, 2012). Besides lncRNA627.1/miR-29a-5p/EDAR, we also identified other DE mRNAs at E41, such as *BMP3* (Nie et al., 2018), *DKK4* (Mokry and Pisal, 2020), *FGFR2* (Nguyen et al., 2018)*,* and *FRZB* (Hu et al., 2020), which participate in hair placode formation *via* the *Wnt/β-catenin, BMP* and *FGF* signaling pathways (Supplementary Table S2). Although excluded from ceRNA construction, these were differentially expressed between normal and hairless pigs at E41, suggesting these genes may exert a synergistic effect on hair placode formation *via* other regulatory mechanisms.

*EDAR* influences hair placode formation *via* its participation in the EDA/EDAR signaling pathway (Schmidt-Ullrich et al., 2006). Therefore, we constructed an interaction network of EDAR-related genes based on previous reports (Fliniaux et al., 2008; Zhang et al., 2009; Wu et al., 2020); (Figure 5B), and detected the expressions of these genes at E39, E41, E45, E52 and E60 stages of normal and hairless pig embryo skins (Figure 5C). The expression trends of *EDA*, *NF-*κ*B*, *Wnt10b* and *BMP4* were consistent with their expression in HPPCs, in which *EDAR* expression was inhibited by *miR-29a-5p* and lncRNA627.1 (Figures 4C,D). Reportedly, *EDA* (Zhang et al., 2009), *Wnt10b* (Andl et al., 2002), and *NF-*κ*B* (Schmidt-Ullrich et al., 2006) were required for the initiation of hair placodes by the EDA/EDAR signaling pathway, while *BMP4* acted as an inhibitor for EDAR (Rishikaysh et al., 2014). On the other hand, the differences between the expression levels of some *EDAR* related genes (Figure 5C) at E41, were not significant (Figure 5B), and this may be attributed to heterogeneity of skin cells (Takahashi et al., 2020) and spatio-temporal gene expression. Our findings not only revealed the importance of *EDAR* in hair placode formation, but also the role played by multi-gene co-regulation which is exerted *via* participation of the EDAR signaling pathway in hair placode formation. However, further research on molecular communication between the multiple genes involved in co-regulation is needed.

## Data Availability

The datasets presented in this study can be found in online repositories. The names of the repository/repositories and accession number(s) can be found below: https://ngdc.cncb.ac.cn/, CRA004672.

## Abbreviations

BMP2/3/4, bone morphogenetic protein 2/3/4; BMPR1b, bone morphogenetic protein receptor type 1b; ceRNA, competitive endogenous RNA; CTNNB1, catenin beta 1; DAPI, 6-diamidino-2-phenylindole; DE, differential expression; DKK4, dickkopf WNT signaling pathway inhibitor 4; EDA, ectodysplasin A; EDAR, ectodysplasin A receptor; Edu, 5-ethynyl-20-deoxyuridine; E41, the day of embryo 41; HF, hair follicle; HPPCs, hair placode precursor cells; IF, immunofluorescence; LHX2, LIM homeobox 2; lncRNA, long non-coding RNA; miRNA, microRNA; ncRNA, non-coding RNA; Pc, hair placode; WB, Western blot; WGCNA, weighted gene co-expression network analysis; Wnt10b, Wnt family member 10b.

## References

[B1] Anders S. Huber W. (2010). Differential Expression Analysis for Sequence Count Data. Genome Biol. 11 (10), R106. 10.1186/gb-2010-11-10-r106 20979621 PMC3218662

[B2] Andl T. Botchkareva N. V. (2015). MicroRNAs (miRNAs) in the Control of HF Development and Cycling: the Next Frontiers in Hair Research. Exp. Dermatol 24 (11), 821–826. 10.1111/exd.12785 26121602 PMC5721351

[B3] Andl T. Murchison E. P. Liu F. Zhang Y. Yunta-Gonzalez M. Tobias J. W. (2006). The miRNA-Processing Enzyme Dicer Is Essential for the Morphogenesis and Maintenance of Hair Follicles. Curr. Biol. 16 (10), 1041–1049. 10.1016/j.cub.2006.04.005 16682203 PMC2996092

[B4] Andl T. Reddy S. T. Gaddapara T. Millar S. E. (2002). WNT Signals Are Required for the Initiation of Hair Follicle Development. Dev. Cell 2 (5), 643–653. 10.1016/s1534-5807(02)00167-3 12015971

[B5] Cai B. Wang X. Liu H. Ma S. Zhang K. Zhang Y. (2019). Up-regulated lncRNA5322 Elevates MAPK1 to Enhance Proliferation of Hair Follicle Stem Cells as a ceRNA of microRNA-19b-3p. Cell Cycle 18 (14), 1588–1600. 10.1080/15384101.2019.1624111 31203719 PMC6619929

[B6] Chen D. Jarrell A. Guo C. Lang R. Atit R. (2012). Dermal β-catenin Activity in Response to Epidermal Wnt Ligands Is Required for Fibroblast Proliferation and Hair Follicle Initiation. Development 139 (8), 1522–1533. 10.1242/dev.076463 22434869 PMC3308184

[B7] Chen S. Huang T. Zhou Y. Han Y. Xu M. Gu J. (2017). AfterQC: Automatic Filtering, Trimming, Error Removing and Quality Control for Fastq Data. BMC Bioinforma. 18 (Suppl. 3), 80. 10.1186/s12859-017-1469-3 PMC5374548 28361673

[B8] Choi B. (2018). Hair-Growth Potential of Ginseng and its Major Metabolites: A Review on its Molecular Mechanisms. Ijms 19 (9), 2703. 10.3390/ijms19092703 PMC6163201 30208587

[B9] Duverger O. Morasso M. I. (2009). Epidermal Patterning and Induction of Different Hair Types during Mouse Embryonic Development. Birth Defect Res. C 87 (3), 263–272. 10.1002/bdrc.20158 PMC2995294 19750518

[B10] Duverger O. Morasso M. I. (2014). To Grow or Not to Grow: Hair Morphogenesis and Human Genetic Hair Disorders. Seminars Cell & Dev. Biol. 25-26, 22–33. 10.1016/j.semcdb.2013.12.006 PMC3988237 24361867

[B11] Finn R. D. Coggill P. Eberhardt R. Y. Eddy S. R. Mistry J. Mitchell A. L. (2016). The Pfam Protein Families Database: towards a More Sustainable Future. Nucleic Acids Res. 44 (D1), D279–D285. 10.1093/nar/gkv1344 26673716 PMC4702930

[B12] Fliniaux I. Mikkola M. L. Lefebvre S. Thesleff I. (2008). Identification of Dkk4 as a Target of Eda-A1/Edar Pathway Reveals an Unexpected Role of Ectodysplasin as Inhibitor of Wnt Signalling in Ectodermal Placodes. Dev. Biol. 320 (1), 60–71. 10.1016/j.ydbio.2008.04.023 18508042

[B13] Friedländer M. R. Mackowiak S. D. Li N. Chen W. Rajewsky N. (2012). miRDeep2 Accurately Identifies Known and Hundreds of Novel microRNA Genes in Seven Animal Clades. Nucleic Acids Res. 40 (1), 37–52. 10.1093/nar/gkr688 21911355 PMC3245920

[B14] Gao Y. Wang X. Yan H. Zeng J. Ma S. Niu Y. (2016). Comparative Transcriptome Analysis of Fetal Skin Reveals Key Genes Related to Hair Follicle Morphogenesis in Cashmere Goats. PLoS One 11 (3), e0151118. 10.1371/journal.pone.0151118 26959817 PMC4784850

[B15] Gay D. L. Yang C.-C. Plikus M. V. Ito M. Rivera C. Treffeisen E. (2015). CD133 Expression Correlates with Membrane Beta-Catenin and E-Cadherin Loss from Human Hair Follicle Placodes during Morphogenesis. J. Investigative Dermatology 135 (1), 45–55. 10.1038/jid.2014.292 PMC4465595 25010141

[B16] Ge M. Liu C. Li L. Lan M. Yu Y. Gu L. (2019). miR-29a/b1 Inhibits Hair Follicle Stem Cell Lineage Progression by Spatiotemporally Suppressing WNT and BMP Signaling. Cell Rep. 29 (8), 2489–2504. 10.1016/j.celrep.2019.10.062 31747615

[B17] Guo L. Huang X. Liang P. Zhang P. Zhang M. Ren L. (2018). Role of XIST/miR-29a/LIN28A Pathway in Denatured Dermis and Human Skin Fibroblasts (HSFs) after Thermal Injury. J Cell. Biochem. 119 (2), 1463–1474. 10.1002/jcb.26307 28771809

[B18] Han W. Yang F. Wu Z. Guo F. Zhang J. Hai E. (2020). Inner Mongolian Cashmere Goat Secondary Follicle Development Regulation Research Based on mRNA-miRNA Co-analysis. Sci. Rep. 10 (1), 4519. 10.1038/s41598-020-60351-5 32161290 PMC7066195

[B19] Hu X. Zhang X. Liu Z. Li S. Zheng X. Nie Y. (2020). Exploration of Key Regulators Driving Primary Feather Follicle Induction in Goose Skin. Gene 731, 144338. 10.1016/j.gene.2020.144338 31923576

[B20] Jaskoll T. Zhou Y.-M. Trump G. Melnick M. (2003). Ectodysplasin Receptor-Mediated Signaling Is Essential for Embryonic Submandibular Salivary Gland Development. Anat. Rec. 271A (2), 322–331. 10.1002/ar.a.10045 12629675

[B21] Jiang Y. Jiang Y. Zhang H. Mei M. Song H. Ma X. (2019). A Mutation in MAP2 Is Associated with Prenatal Hair Follicle Density. FASEB J. 33 (12), 14479–14490. 10.1096/fj.201901187R 31751154

[B22] Jiang Y. Zou Q. Liu B. Li S. Wang Y. Liu T. (2021). Atlas of Prenatal Hair Follicle Morphogenesis Using the Pig as a Model System. Front. Cell Dev. Biol. 9, 721979. 10.3389/fcell.2021.721979 34692680 PMC8529045

[B23] Kang Y.-J. Yang D.-C. Kong L. Hou M. Meng Y.-Q. Wei L. (2017). CPC2: a Fast and Accurate Coding Potential Calculator Based on Sequence Intrinsic Features. Nucleic Acids Res. 45 (W1), W12–W16. 10.1093/nar/gkx428 28521017 PMC5793834

[B24] Kim D. Paggi J. M. Park C. Bennett C. Salzberg S. L. (2019). Graph-based Genome Alignment and Genotyping with HISAT2 and HISAT-Genotype. Nat. Biotechnol. 37 (8), 907–915. 10.1038/s41587-019-0201-4 31375807 PMC7605509

[B25] Lee J. Bӧscke R. Tang P.-C. Hartman B. H. Heller S. Koehler K. R. (2018). Hair Follicle Development in Mouse Pluripotent Stem Cell-Derived Skin Organoids. Cell Rep. 22 (1), 242–254. 10.1016/j.celrep.2017.12.007 29298425 PMC5806130

[B26] Lee J. Koehler K. R. (2021). Skin Organoids: A New Human Model for Developmental and Translational Research. Exp. Dermatol 30 (4), 613–620. 10.1111/exd.14292 33507537 PMC8265774

[B27] Lee J. Rabbani C. C. Gao H. Steinhart M. R. Woodruff B. M. Pflum Z. E. (2020). Hair-bearing Human Skin Generated Entirely from Pluripotent Stem Cells. Nature 582 (7812), 399–404. 10.1038/s41586-020-2352-3 32494013 PMC7593871

[B28] Lin M. F. Jungreis I. Kellis M. (2011). PhyloCSF: a Comparative Genomics Method to Distinguish Protein Coding and Non-coding Regions. Bioinformatics 27 (13), i275–i282. 10.1093/bioinformatics/btr209 21685081 PMC3117341

[B29] Liu X. Zhang P. Ji K. Zhang J. Yang S. Du B. (2018a). Cyclin-dependent Kinase 5 Regulates MAPK/ERK Signaling in the Skin of Mice. Acta Histochem. 120 (1), 15–21. 10.1016/j.acthis.2017.10.009 29132690

[B30] Liu Z. Yang F. Zhao M. Ma L. Li H. Xie Y. (2018b). The Intragenic mRNA-microRNA Regulatory Network during Telogen-Anagen Hair Follicle Transition in the Cashmere Goat. Sci. Rep. 8 (1), 14227. 10.1038/s41598-018-31986-2 30242252 PMC6155037

[B31] Love M. I. Huber W. Anders S. (2014). Moderated Estimation of Fold Change and Dispersion for RNA-Seq Data with DESeq2. Genome Biol. 15 (12), 550. 10.1186/s13059-014-0550-8 25516281 PMC4302049

[B32] Luo Z. Dou J. Xie F. Lu J. Han Q. Zhou X. (2021). miR -203a-3p Promotes Loureirin A-Induced Hair Follicle Stem Cells Differentiation by Targeting Smad1. Anat. Rec. 304 (3), 531–540. 10.1002/ar.24480 32589363

[B33] Ma S. Wang Y. Zhou G. Ding Y. Yang Y. Wang X. (2019). Synchronous Profiling and Analysis of mRNAs and ncRNAs in the Dermal Papilla Cells from Cashmere Goats. BMC Genomics 20 (1), 512. 10.1186/s12864-019-5861-4 31221080 PMC6587304

[B34] Ma T. Li J. Li J. Wu S. Ba X. Jiang H. (2021). Expression of miRNA-203 and its Target Gene in Hair Follicle Cycle Development of Cashmere Goat. Cell Cycle 20 (2), 204–210. 10.1080/15384101.2020.1867789 33427027 PMC7889112

[B35] Mohri Y. Kato S. Umezawa A. Okuyama R. Nishimori K. (2008). Impaired Hair Placode Formation with Reduced Expression of Hair Follicle-Related Genes in Mice Lacking Lgr4. Dev. Dyn. 237 (8), 2235–2242. 10.1002/dvdy.21639 18651655

[B36] Mokry J. Pisal R. (2020). Development and Maintenance of Epidermal Stem Cells in Skin Adnexa. Ijms 21 (24), 9736. 10.3390/ijms21249736 PMC7766199 33419358

[B37] Mustonen T. Ilmonen M. Pummila M. Kangas A. T. Laurikkala J. Jaatinen R. (2004). Ectodysplasin A1 Promotes Placodal Cell Fate during Early Morphogenesis of Ectodermal Appendages. Development 131 (20), 4907–4919. 10.1242/dev.01377 15371307

[B38] Nguyen M. B. Cohen I. Kumar V. Xu Z. Bar C. Dauber-Decker K. L. (2018). FGF Signalling Controls the Specification of Hair Placode-Derived SOX9 Positive Progenitors to Merkel Cells. Nat. Commun. 9 (1), 2333. 10.1038/s41467-018-04399-y 29899403 PMC5998134

[B39] Nie Y. Li S. Zheng X. Chen W. Li X. Liu Z. (2018). Transcriptome Reveals Long Non-coding RNAs and mRNAs Involved in Primary Wool Follicle Induction in Carpet Sheep Fetal Skin. Front. Physiol. 9, 446. 10.3389/fphys.2018.00446 29867522 PMC5968378

[B40] Patel R. K. Jain M. (2012). NGS QC Toolkit: a Toolkit for Quality Control of Next Generation Sequencing Data. PLoS One 7 (2), e30619. 10.1371/journal.pone.0030619 22312429 PMC3270013

[B41] Pei G. Chen L. Zhang W. (2017). WGCNA Application to Proteomic and Metabolomic Data Analysis. Methods Enzymol. 585, 135–158. 10.1016/bs.mie.2016.09.016 28109426

[B42] Pertea M. Kim D. Pertea G. M. Leek J. T. Salzberg S. L. (2016). Transcript-level Expression Analysis of RNA-Seq Experiments with HISAT, StringTie and Ballgown. Nat. Protoc. 11 (9), 1650–1667. 10.1038/nprot.2016.095 27560171 PMC5032908

[B43] Quinlan A. R. Hall I. M. (2010). BEDTools: a Flexible Suite of Utilities for Comparing Genomic Features. Bioinformatics 26 (6), 841–842. 10.1093/bioinformatics/btq033 20110278 PMC2832824

[B44] Ravasz E. Somera A. L. Mongru D. A. Oltvai Z. N. Barabási A.-L. (2002). Hierarchical Organization of Modularity in Metabolic Networks. Science 297 (5586), 1551–1555. 10.1126/science.1073374 12202830

[B45] Rishikaysh P. Dev K. Diaz D. Qureshi W. Filip S. Mokry J. (2014). Signaling Involved in Hair Follicle Morphogenesis and Development. Ijms 15 (1), 1647–1670. 10.3390/ijms15011647 24451143 PMC3907891

[B46] Salmena L. Poliseno L. Tay Y. Kats L. Pandolfi P. P. (2011). A ceRNA Hypothesis: the Rosetta Stone of a Hidden RNA Language? Cell 146 (3), 353–358. 10.1016/j.cell.2011.07.014 21802130 PMC3235919

[B47] Saxena N. Mok K. W. Rendl M. (2019). An Updated Classification of Hair Follicle Morphogenesis. Exp. Dermatol 28 (4), 332–344. 10.1111/exd.13913 30887615 PMC7137758

[B48] Schmidt-Ullrich R. Tobin D. J. Lenhard D. Schneider P. Paus R. Scheidereit C. (2006). NF-κB Transmits Eda A1/EdaR Signalling to Activate Shh and Cyclin D1 Expression, and Controls Post-initiation Hair Placode Down Growth. Development 133 (6), 1045–1057. 10.1242/dev.02278 16481354

[B49] Schneider M. R. Schmidt-Ullrich R. Paus R. (2009). The Hair Follicle as a Dynamic Miniorgan. Curr. Biol. 19 (3), R132–R142. 10.1016/j.cub.2008.12.005 19211055

[B50] Sennett R. Rendl M. (2012). Mesenchymal-epithelial Interactions during Hair Follicle Morphogenesis and Cycling. Seminars Cell & Dev. Biol. 23 (8), 917–927. 10.1016/j.semcdb.2012.08.011 PMC3496047 22960356

[B51] Shannon P. Markiel A. Ozier O. Baliga N. S. Wang J. T. Ramage D. (2003). Cytoscape: a Software Environment for Integrated Models of Biomolecular Interaction Networks. Genome Res. 13 (11), 2498–2504. 10.1101/gr.1239303 14597658 PMC403769

[B52] St-Jacques B. Dassule H. R. Karavanova I. Botchkarev V. A. Li J. Danielian P. S. (1998). Sonic Hedgehog Signaling Is Essential for Hair Development. Curr. Biol. 8 (19), 1058–1069. 10.1016/s0960-9822(98)70443-9 9768360

[B53] Sun L. Luo H. Bu D. Zhao G. Yu K. Zhang C. (2013). Utilizing Sequence Intrinsic Composition to Classify Protein-Coding and Long Non-coding Transcripts. Nucleic Acids Res. 41 (17), e166. 10.1093/nar/gkt646 23892401 PMC3783192

[B54] Takahashi R. Grzenda A. Allison T. F. Rawnsley J. Balin S. J. Sabri S. (2020). Defining Transcriptional Signatures of Human Hair Follicle Cell States. J. Investigative Dermatology 140 (4), 764–773. e764. 10.1016/j.jid.2019.07.726 PMC7093259 31676413

[B55] Tam S. Tsao M.-S. McPherson J. D. (2015). Optimization of miRNA-Seq Data Preprocessing. Briefings Bioinforma. 16 (6), 950–963. 10.1093/bib/bbv019 PMC4652620 25888698

[B56] Tay Y. Rinn J. Pandolfi P. P. (2014). The Multilayered Complexity of ceRNA Crosstalk and Competition. Nature 505 (7483), 344–352. 10.1038/nature12986 24429633 PMC4113481

[B57] Teta M. Choi Y. S. Okegbe T. Wong G. Tam O. H. Chong M. M. W. (2012). Inducible Deletion of Epidermal Dicer and Drosha Reveals Multiple Functions for miRNAs in Postnatal Skin. Development 139 (8), 1405–1416. 10.1242/dev.070920 22434867 PMC3308177

[B58] Tomann P. Paus R. Millar S. E. Scheidereit C. Schmidt-Ullrich R. (2016). LHX2 Is a Direct NF-Κb Target Gene that Promotes Primary Hair Follicle Placode Down-Growth. Development 143 (9), 1512–1522. 10.1242/dev.130898 26952977 PMC6514410

[B59] Vishlaghi N. Lisse T. S. (2020). Dicer- and Bulge Stem Cell-dependent MicroRNAs during Induced Anagen Hair Follicle Development. Front. Cell Dev. Biol. 8, 338. 10.3389/fcell.2020.00338 32478074 PMC7240072

[B60] Wang A. B. Zhang Y. V. Tumbar T. (2017a). Gata6 Promotes Hair Follicle Progenitor Cell Renewal by Genome Maintenance during Proliferation. EMBO J. 36 (1), 61–78. 10.15252/embj.201694572 27908934 PMC5210152

[B61] Wang S. Ge W. Luo Z. Guo Y. Jiao B. Qu L. (2017b). Integrated Analysis of Coding Genes and Non-coding RNAs during Hair Follicle Cycle of Cashmere Goat (*Capra hircus*). BMC Genomics 18 (1), 767. 10.1186/s12864-017-4145-0 29020916 PMC5637055

[B62] Wu Z. Wang Y. Han W. Yang K. Hai E. Ma R. (2020). EDA and EDAR Expression at Different Stages of Hair Follicle Development in Cashmere Goats and Effects on Expression of Related Genes. Arch. Anim. Breed. 63 (2), 461–470. 10.5194/aab-63-461-2020 33473371 PMC7810227

[B63] Yan H. Tang H. Qiu W. Tan R. Zhang W. Yang G. (2019b). A New Dynamic Culture Device Suitable for Rat Skin Culture. Cell Tissue Res. 375 (3), 723–731. 10.1007/s00441-018-2945-4 30392145

[B64] Yan H. Gao Y. Ding Q. Liu J. Li Y. Jin M. (2019a). Exosomal Micro RNAs Derived from Dermal Papilla Cells Mediate Hair Follicle Stem Cell Proliferation and Differentiation. Int. J. Biol. Sci. 15 (7), 1368–1382. 10.7150/ijbs.33233 31337968 PMC6643152

[B65] Yi R. O'Carroll D. Pasolli H. A. Zhang Z. Dietrich F. S. Tarakhovsky A. (2006). Morphogenesis in Skin Is Governed by Discrete Sets of Differentially Expressed microRNAs. Nat. Genet. 38 (3), 356–362. 10.1038/ng1744 16462742

[B66] Yi R. Pasolli H. A. Landthaler M. Hafner M. Ojo T. Sheridan R. (2009). DGCR8-dependent microRNA Biogenesis Is Essential for Skin Development. Proc. Natl. Acad. Sci. U.S.A. 106 (2), 498–502. 10.1073/pnas.0810766105 19114655 PMC2626731

[B67] Yue Y. Guo T. Yuan C. Liu J. Guo J. Feng R. (2016). Integrated Analysis of the Roles of Long Noncoding RNA and Coding RNA Expression in Sheep (*Ovis aries*) Skin during Initiation of Secondary Hair Follicle. PLoS One 11 (6), e0156890. 10.1371/journal.pone.0156890 27276011 PMC4898689

[B68] Zhang Y. Tomann P. Andl T. Gallant N. M. Huelsken J. Jerchow B. (2009). Reciprocal Requirements for EDA/EDAR/NF-κB and Wnt/β-Catenin Signaling Pathways in Hair Follicle Induction. Dev. Cell 17 (1), 49–61. 10.1016/j.devcel.2009.05.011 19619491 PMC2859042

[B69] Zhao B. Chen Y. Yang N. Chen Q. Bao Z. Liu M. (2019). miR-218-5p Regulates Skin and Hair Follicle Development through Wnt/β-Catenin Signaling Pathway by Targeting SFRP2. J. Cell. Physiology 234 (11), 20329–20341. 10.1002/jcp.28633 30953362

[B70] Zhao R. Li J. Liu N. Li H. Liu L. Yang F. (2020). Transcriptomic Analysis Reveals the Involvement of lncRNA-miRNA-mRNA Networks in Hair Follicle Induction in Aohan Fine Wool Sheep Skin. Front. Genet. 11, 590. 10.3389/fgene.2020.00590 33117415 PMC7528302

[B71] Zhou J. Zhang X. Liang P. Ren L. Zeng J. Zhang M. (2016). Protective Role of microRNA-29a in Denatured Dermis and Skin Fibroblast Cells after Thermal Injury. Biol. Open 5 (3), 211–219. 10.1242/bio.014910 26794609 PMC4810739

[B72] Zhou L. Zhang X. Paus R. Lu Z. (2018). The Renaissance of Human Skin Organ Culture: A Critical Reappraisal. Differentiation 104, 22–35. 10.1016/j.diff.2018.10.002 30391646

[B73] Zhu N. Huang K. Liu Y. Zhang H. Lin E. Zeng Y. (20182018). miR-195-5p Regulates Hair Follicle Inductivity of Dermal Papilla Cells by Suppressing Wnt/β-Catenin Activation. BioMed Res. Int. 2018, 1–13. 10.1155/2018/4924356 PMC5937601 29850524

[B74] Zhu N. Lin E. Zhang H. Liu Y. Cao G. Fu C. (2020). LncRNA H19 Overexpression Activates Wnt Signaling to Maintain the Hair Follicle Regeneration Potential of Dermal Papilla Cells. Front. Genet. 11, 694. 10.3389/fgene.2020.00694 32849769 PMC7417632

