# Supplementary material for: miR-29a-5p Inhibits Prenatal Hair Placode Formation Through Targeting EDAR by ceRNA Regulatory Network
Source: Front Cell Dev Biol. 2022 May 12;10:902026. doi: 10.3389/fcell.2022.902026 (PMC9133881; doi:10.3389/fcell.2022.902026)
Supplement: Supplementary file 1 [file DataSheet1.docx]

Supplementary Material

# Supplementary Figures and Tables

## Supplementary Figures


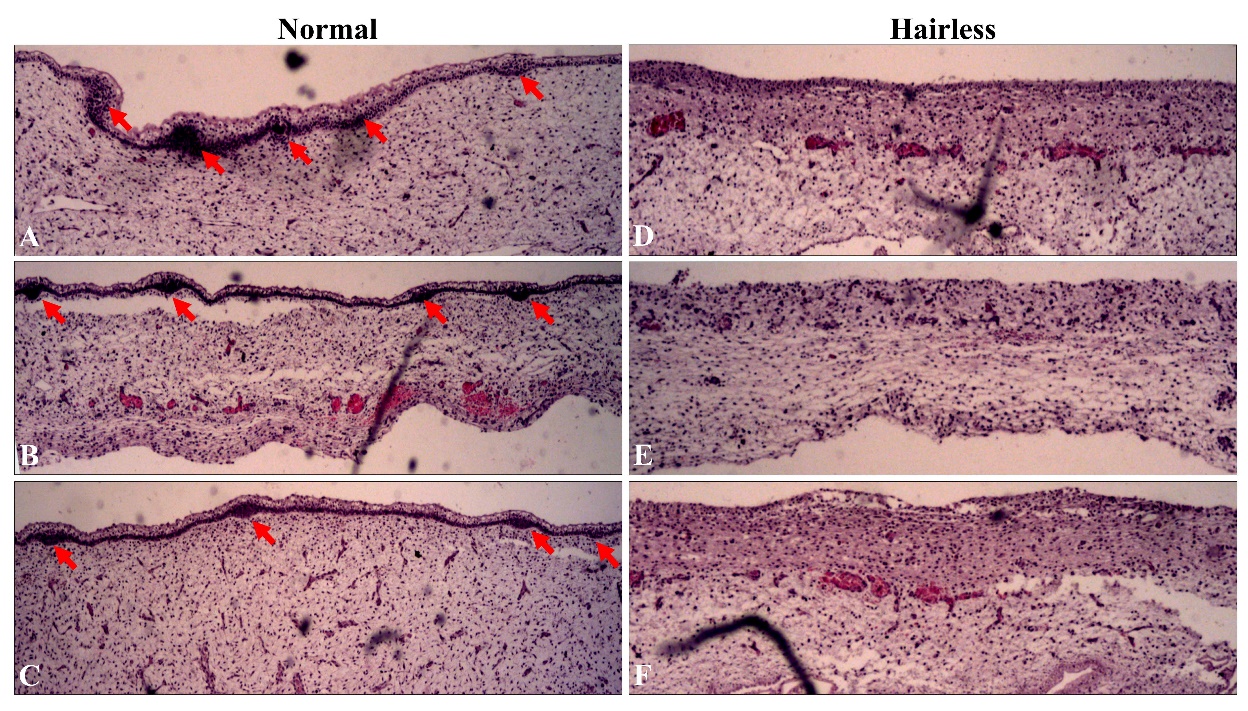


**Supplementary Figure 1 The phenotype detection of each sample in normal and hairless groups at E41.** The red arrow represents hair placode in epidermal


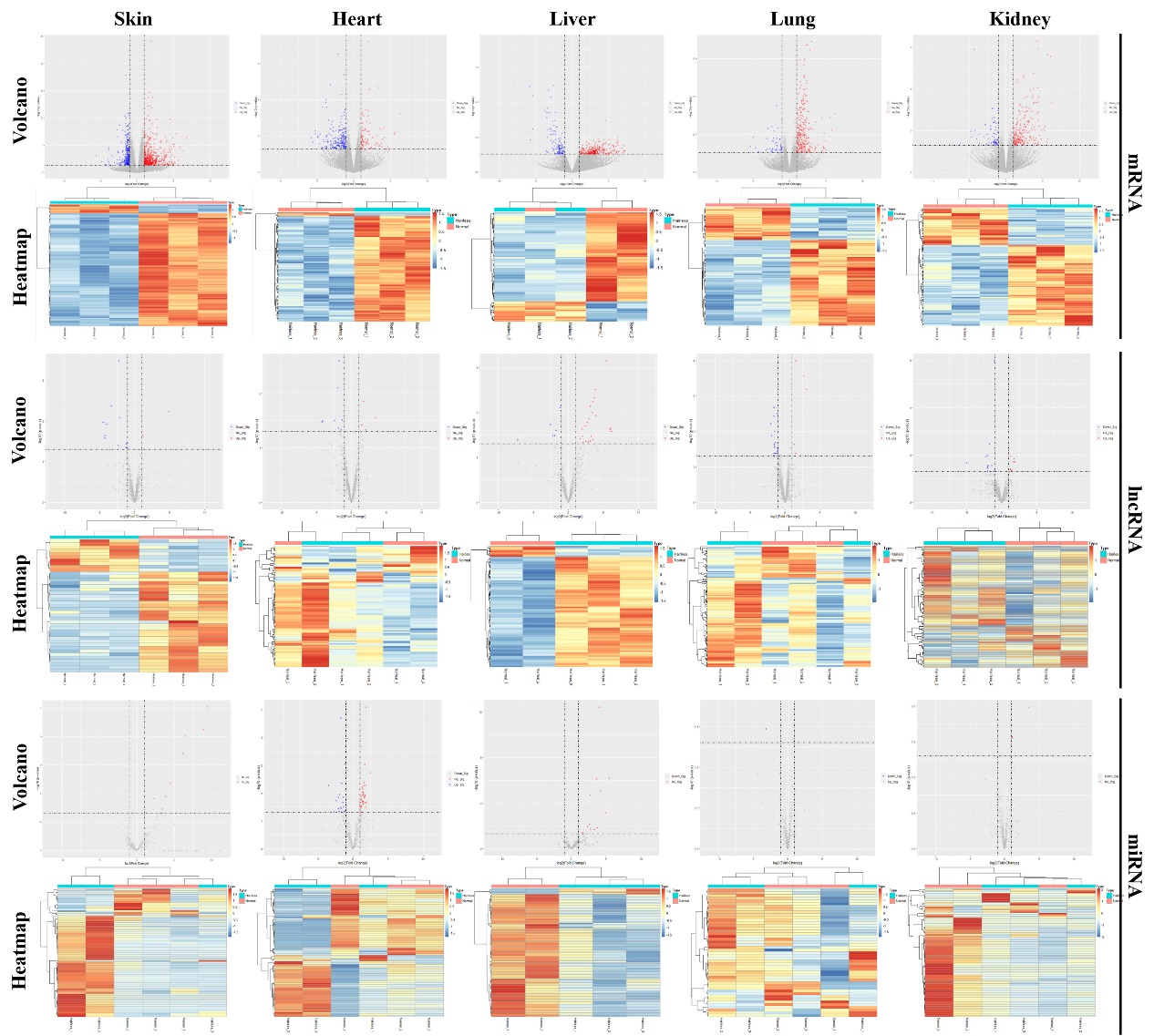


**Supplementary Figure 2 Differentially Expression mRNAs, miRNAs and lncRNAs between hairless and normal pig embryos in skin, heart, liver, lung, kidney.**

Volcano plots for the up- and down-regulated mRNAs **(A)**, miRNAs **(E)** and lncRNAs **(J)** at E41 skin between normal and hairless pigs;


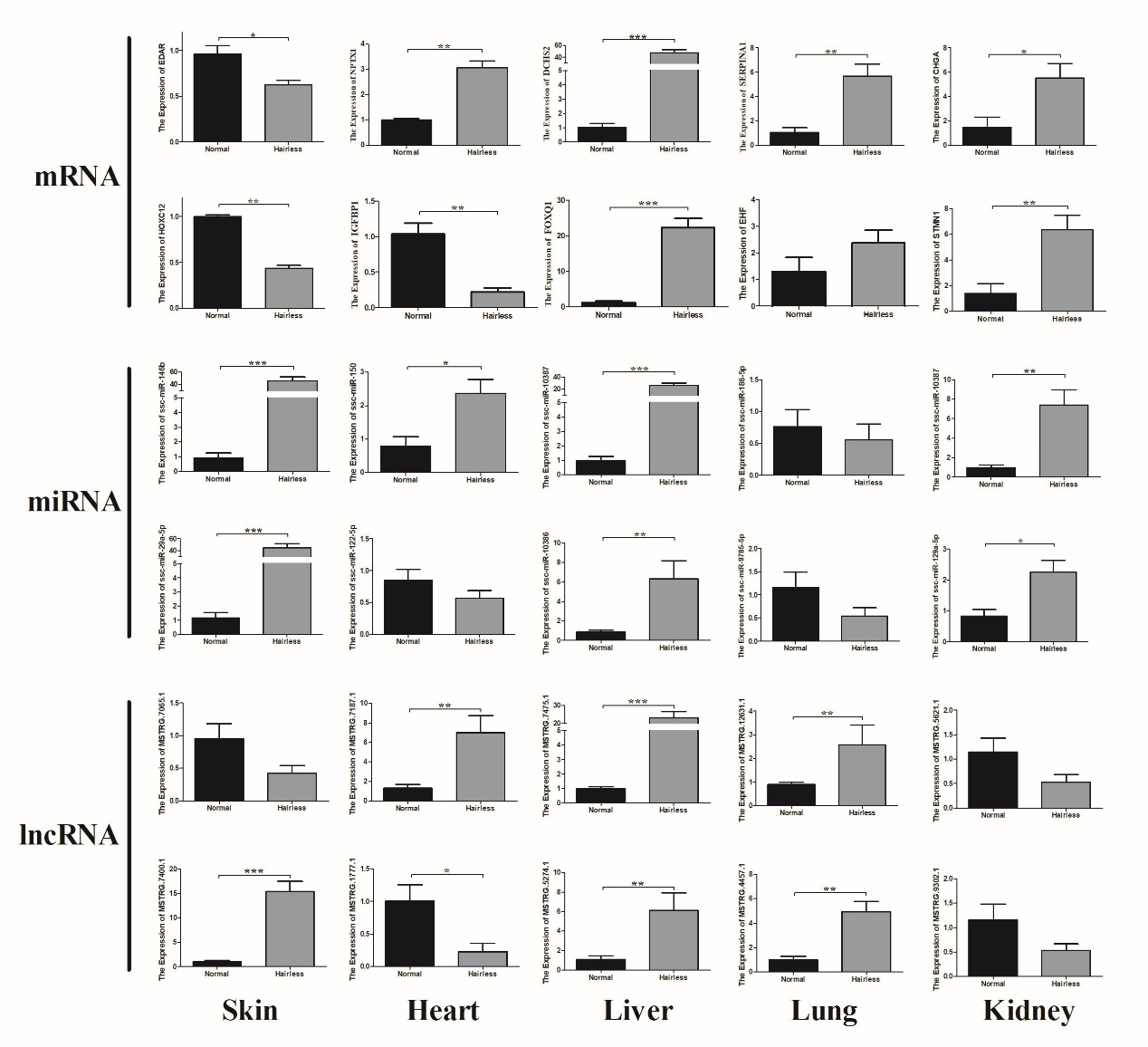


**Supplementary Figure 3 Validation of mRNA, miRNA and lncRNA differential expression results between hairless and normal pigs in E41.**

Two DE mRNAs, miRNAs and lncRNAs were selected randomly for each tissue (skin, heart, liver, lung and kidney). Error bars indicate the mean±SD of triplicate experiments. **P* < 0.05; ***P* < 0.01, ****P* < 0.001.


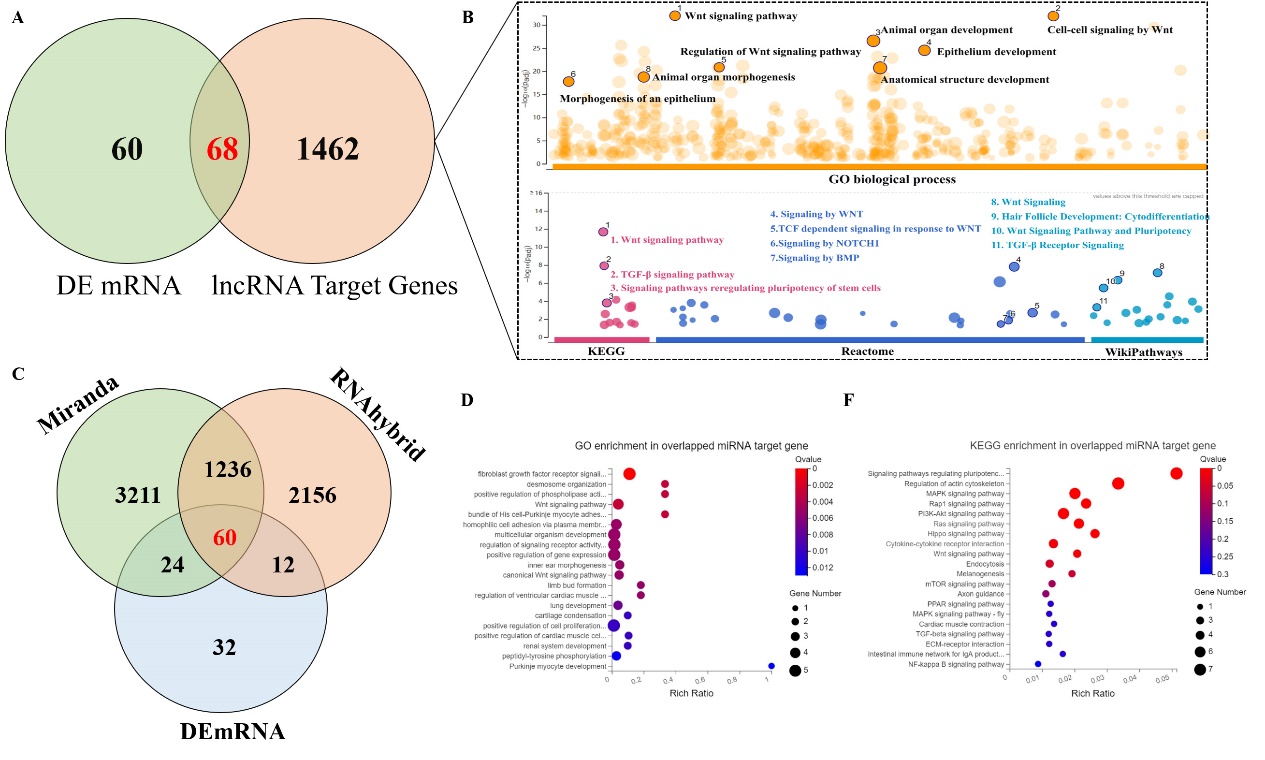


**Supplementary Figure 4 ncRNA target genes screening and GO and KEGG enrichment analysis.**

**(A)** Venn diagram for 68 overlapped genes between 128 DE mRNA and 1530 DE lncRNA target genes; **(B)** GO and KEGG enrichment analysis for 68 overlapped genes; **(C)** Venn diagram for 60 overlapped genes between DE miRNA target genes and 128 DE mRNA; (**D)** Top 20 terms of GO enrichment in overlapped miRNA genes; **(F)** Top 20 terms of KEGG enrichment in overlapped miRNA genes;


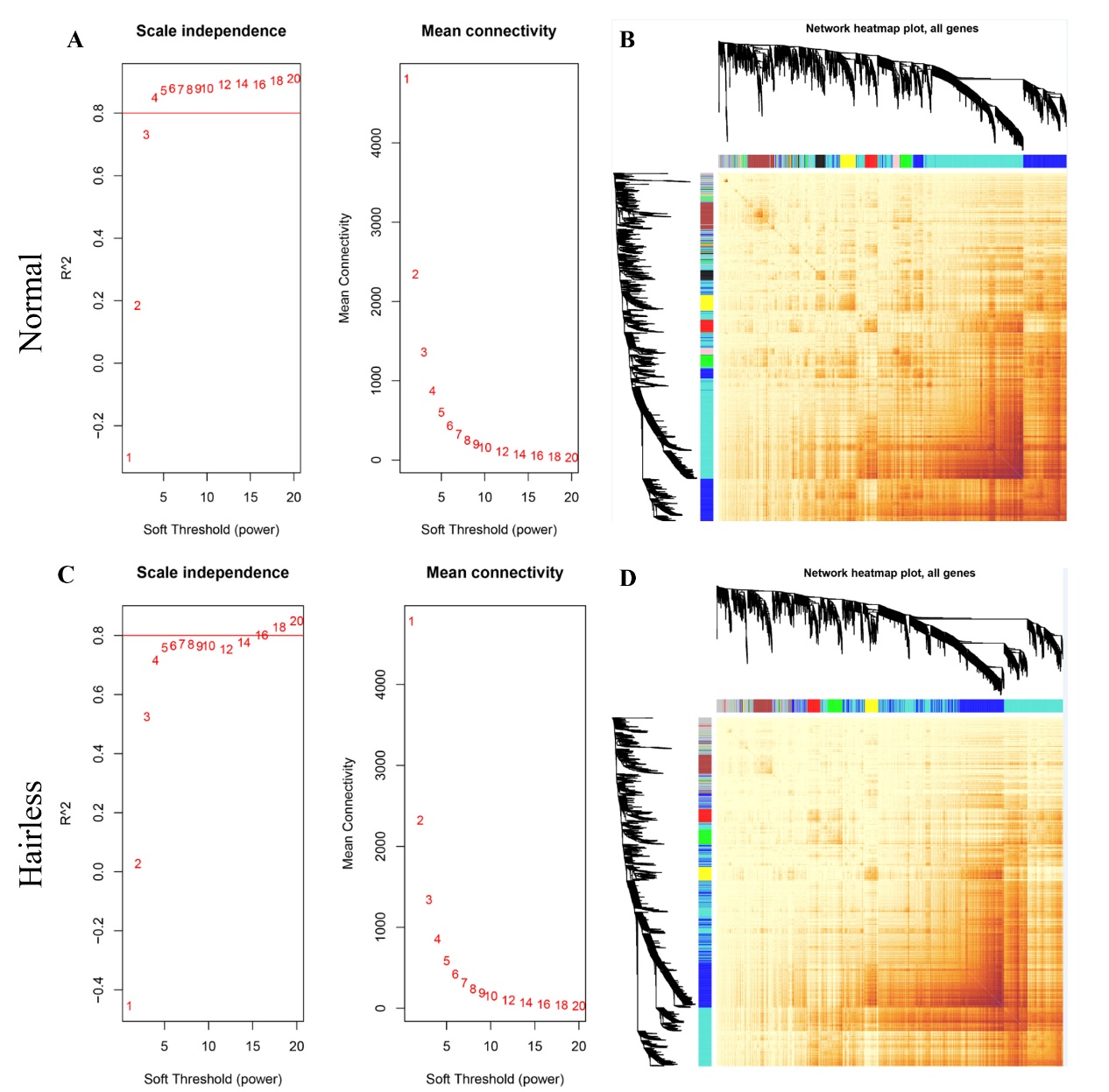


**Supplementary Figure 5 WGCNA for hairless and normal pig skin in different periods.**

**(A, C)** Analysis of network topology for various soft thresholding powers of Normal**(A)** and Hairless**(C)** pig skin in different periods. The left panel shows the scale-free fit index (y-axis) as a function of the soft-thresholding power (x-axis). The right panel displays the mean connectivity (degree, y-axis) as a function of the soft-thresholding power (x-axis). **(B，D)** Visualizing the gene network using a heatmap plot of Normal**(B)** and Hairless**(D)** pig skin in different periods. Light color represents low overlap and progressively darker red color represents higher overlap. Blocks of darker colors along the diagonal are the modules. The gene dendrogram and module assignment are also shown along the left side and the top.


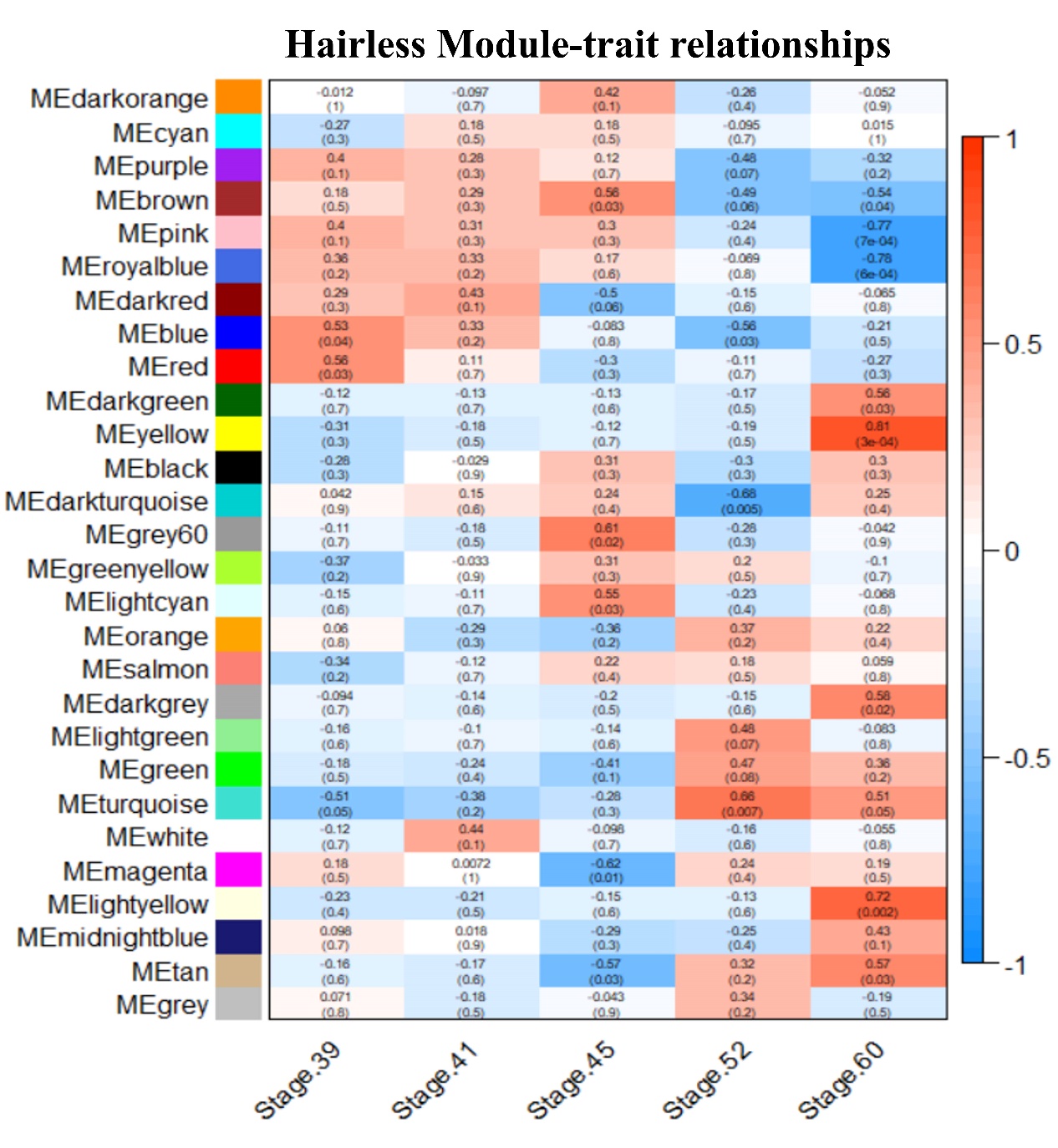


**Supplementary Figure 6 Module-trait associations of hairless pig skin during different periods.**

Each row corresponds to a module epigene, while each column corresponds to a period. Each cell contains the corresponding correlation and p-value; The table is color-coded by correlation according to the color legend;


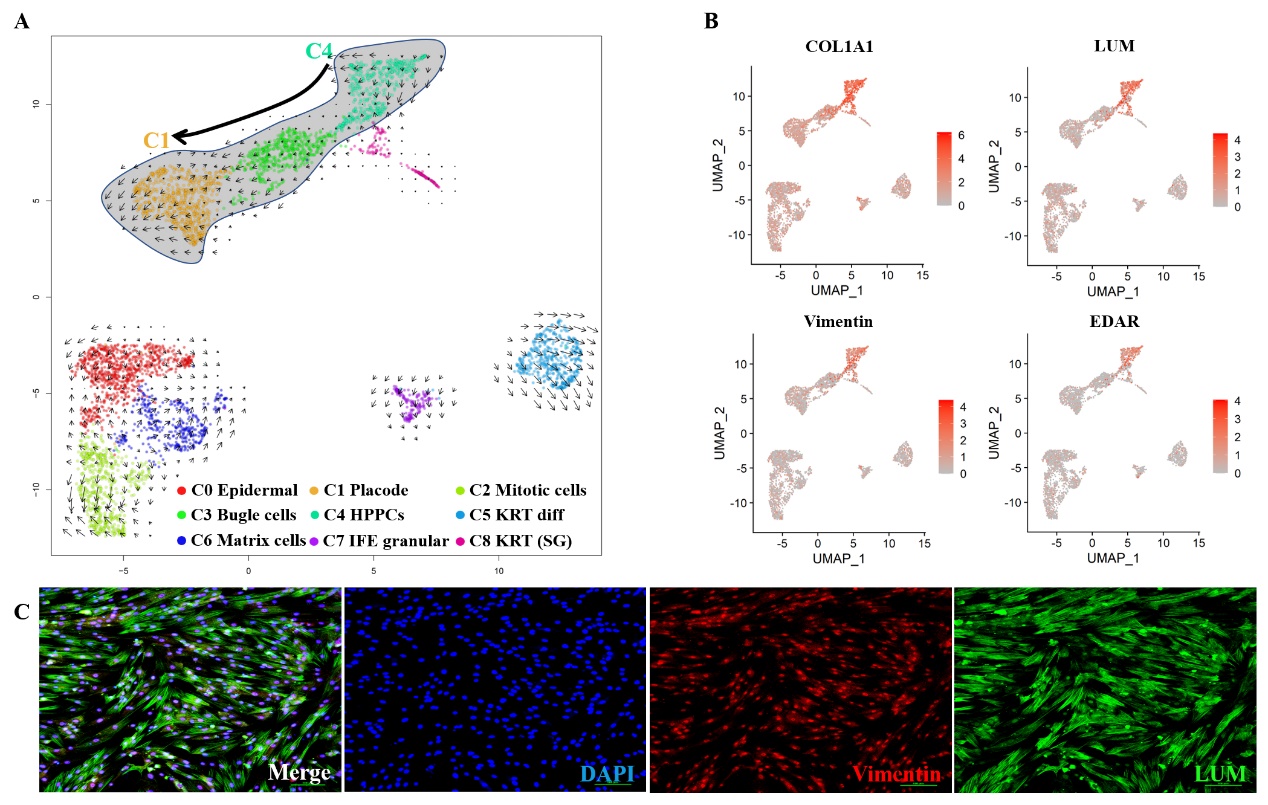


**Supplementary Figure 7 Precursor cell of hair placode (HPPCs) were identified by single cell transcriptome sequencing in embryonic**

**(A)** Characterization of nine epidermis cell subclassify in the UMAP plot. Each point represents one single cell and cells in the same cluster represents high similarity in transcriptome profile. The black arrows represent cell differentiation trajectory of different cell subtypes while black shaded area represents the differentiation trajectory of HPPCs (Cluster 4, C4) to hair placode (Cluster 1, C1); **(B)** Visualization of fibroblast canonical maker gene (*COL1A1, LUM*, and *Vimentin*) and *EDAR* expression in the UMAP plot of HPPCs; **(C)** Dual Immunofluorescence analysis of *LUM*^+^ (green) with *Vimentin*^+^ (red) expression in HPPCs. Scale bars, 100 μm.

## Supplementary Table

**Supplementary Table 1 Summary of mRNA and lncRNA sequencing for each sample**

| **Sample ID** | **Type** | **Raw Reads** | **Clean Reads** | **Raw Base(G)** | **Clean Base(G)** | **Effective Rate(%)** | **Error Rate(%)** | **Q20(%)** | **Q30(%)** | **GC Content(%)** |
| --- | --- | --- | --- | --- | --- | --- | --- | --- | --- | --- |
| **E41Heart1** | mRNA/lncRNA | 25,891,301 | 24,808,808 | 7.77 | 7.44 | 95.82 | 0.03 | 97.91 | 94.3 | 53.04 |
| **E41Heart2** | mRNA/lncRNA | 19,924,168 | 19,282,809 | 5.98 | 5.78 | 96.78 | 0.02 | 98.36 | 95.35 | 51.39 |
| **E41Heart3** | mRNA/lncRNA | 19,860,615 | 19,286,538 | 5.96 | 5.79 | 97.11 | 0.02 | 98.41 | 95.55 | 52.22 |
| **E41Heart4** | mRNA/lncRNA | 20,005,343 | 19,253,535 | 6 | 5.78 | 96.24 | 0.02 | 98.35 | 95.34 | 51.83 |
| **E41Heart6** | mRNA/lncRNA | 25,159,285 | 24,491,686 | 7.55 | 7.35 | 97.35 | 0.03 | 97.96 | 94.27 | 51.79 |
| **E41Heart5** | mRNA/lncRNA | 13,852,500 | 13,687,901 | 4.16 | 4.11 | 98.81 | 0.03 | 97.75 | 93.95 | 51.26 |
| **E41Liver1** | mRNA/lncRNA | 21,018,186 | 20,393,502 | 6.31 | 6.12 | 97.03 | 0.03 | 98.16 | 94.77 | 53.73 |
| **E41Liver3** | mRNA/lncRNA | 33,269,532 | 30,337,541 | 9.98 | 9.1 | 91.19 | 0.02 | 98.72 | 95.9 | 49.97 |
| **E41Liver4** | mRNA/lncRNA | 30,495,332 | 29,371,998 | 9.15 | 8.81 | 96.32 | 0.02 | 98.19 | 94.84 | 53.78 |
| **E41Liver6** | mRNA/lncRNA | 30,201,082 | 29,083,318 | 9.06 | 8.72 | 96.3 | 0.02 | 98.54 | 95.7 | 53.67 |
| **E41Liver5** | mRNA/lncRNA | 19,233,908 | 18,473,283 | 5.77 | 5.54 | 96.05 | 0.02 | 98.34 | 95.38 | 52.95 |
| **E41Lung1** | mRNA/lncRNA | 25,844,128 | 25,118,263 | 7.75 | 7.54 | 97.19 | 0.03 | 97.99 | 94.46 | 52.84 |
| **E41Lung2** | mRNA/lncRNA | 23,305,812 | 22,568,744 | 6.99 | 6.77 | 96.84 | 0.03 | 98.03 | 94.54 | 52.5 |
| **E41Lung3** | mRNA/lncRNA | 22,477,063 | 21,974,960 | 6.74 | 6.59 | 97.77 | 0.03 | 97.91 | 94.23 | 52.93 |
| **E41Lung4** | mRNA/lncRNA | 33,288,516 | 32,692,143 | 9.99 | 9.81 | 98.21 | 0.03 | 97.84 | 94.09 | 52.34 |
| **E41Lung6** | mRNA/lncRNA | 25,408,544 | 24,590,400 | 7.62 | 7.38 | 96.78 | 0.02 | 98.32 | 95.35 | 52.36 |
| **E41Lung5** | mRNA/lncRNA | 25,785,423 | 24,799,859 | 7.74 | 7.44 | 96.18 | 0.02 | 98.36 | 95.42 | 52.94 |
| **E41Kidney1** | mRNA/lncRNA | 30,283,695 | 29,029,566 | 9.09 | 8.71 | 95.86 | 0.03 | 97.94 | 94.34 | 54.52 |
| **E41Kidney2** | mRNA/lncRNA | 30,695,922 | 30,077,877 | 9.21 | 9.02 | 97.99 | 0.03 | 97.73 | 93.84 | 52.54 |
| **E41Kidney3** | mRNA/lncRNA | 37,431,320 | 36,581,979 | 11.23 | 10.97 | 97.73 | 0.03 | 97.79 | 94.01 | 53.13 |
| **E41Kidney4** | mRNA/lncRNA | 26,155,461 | 25,414,106 | 7.85 | 7.62 | 97.17 | 0.03 | 97.9 | 94.25 | 52.27 |
| **E41Kidney6** | mRNA/lncRNA | 21,179,472 | 20,594,106 | 6.35 | 6.18 | 97.24 | 0.03 | 97.84 | 94.17 | 52 |
| **E41Kidney5** | mRNA/lncRNA | 24719463 | 24113974 | 7.42 | 7.23 | 97.55 | 0.03 | 98 | 94.45 | 52.77 |
| **E41Skin1** | mRNA/lncRNA | 29178085 | 28715299 | 8.75 | 8.61 | 98.41 | 0.03 | 96.75 | 91.85 | 53.88 |
| **E41Skin2** | mRNA/lncRNA | 28598145 | 28192210 | 8.58 | 8.46 | 98.58 | 0.03 | 96.78 | 91.85 | 52.69 |
| **E41Skin3** | mRNA/lncRNA | 28674343 | 28278503 | 8.6 | 8.48 | 98.62 | 0.03 | 96.79 | 91.9 | 53.83 |
| **E41Skin4** | mRNA/lncRNA | 28276794 | 27888621 | 8.48 | 8.37 | 98.63 | 0.03 | 97.03 | 92.35 | 53.11 |
| **E41Skin5** | mRNA/lncRNA | 30634466 | 30162217 | 9.19 | 9.05 | 98.46 | 0.03 | 96.97 | 92.26 | 53.19 |
| **E41Skin6** | mRNA/lncRNA | 27456705 | 27074141 | 8.24 | 8.12 | 98.61 | 0.03 | 96.68 | 91.65 | 53.72 |
| **E37Skin1** | mRNA | 26939168 | 26557961 | 8.08 | 7.97 | 98.58 | 0.03 | 96.63 | 91.52 | 52.5 |
| **E37Skin2** | mRNA | 26857363 | 26545360 | 8.06 | 7.96 | 98.84 | 0.03 | 97.13 | 92.54 | 52.91 |
| **E37Skin3** | mRNA | 22693778 | 22362188 | 6.81 | 6.71 | 98.54 | 0.03 | 96.05 | 89.97 | 52.86 |
| **E39Skin1** | mRNA | 23765699 | 23508646 | 7.13 | 7.05 | 98.92 | 0.03 | 96.69 | 91.65 | 53.35 |
| **E39Skin2** | mRNA | 29203680 | 28766566 | 8.76 | 8.63 | 98.5 | 0.03 | 96.78 | 91.89 | 53.5 |
| **E39Skin3** | mRNA | 28797964 | 28344010 | 8.64 | 8.5 | 98.42 | 0.03 | 96.91 | 92.07 | 53.09 |
| **E39Skin4** | mRNA | 27652731 | 27200092 | 8.3 | 8.16 | 98.36 | 0.03 | 96.74 | 91.73 | 53.95 |
| **E39Skin5** | mRNA | 28230059 | 27879639 | 8.47 | 8.36 | 98.76 | 0.03 | 97.01 | 92.36 | 53.83 |
| **E39Skin7** | mRNA | 25658014 | 25238205 | 7.7 | 7.57 | 98.36 | 0.03 | 97.09 | 92.53 | 54.62 |
| **E45Skin2** | mRNA | 29139463 | 28703976 | 8.74 | 8.61 | 98.51 | 0.03 | 96.68 | 91.69 | 52.91 |
| **E45Skin3** | mRNA | 24188221 | 23890952 | 7.26 | 7.17 | 98.77 | 0.03 | 96.59 | 91.46 | 53.45 |
| **E45Skin4** | mRNA | 28093226 | 27685246 | 8.43 | 8.31 | 98.55 | 0.03 | 96.87 | 92.06 | 53.74 |
| **E45Skin5** | mRNA | 27554887 | 27180283 | 8.27 | 8.15 | 98.64 | 0.03 | 96.75 | 91.82 | 52.88 |
| **E45Skin11** | mRNA | 30040477 | 29598597 | 9.01 | 8.88 | 98.53 | 0.03 | 96.86 | 92.03 | 53.3 |
| **E45Skin10** | mRNA | 27220694 | 26778128 | 8.17 | 8.03 | 98.37 | 0.03 | 96.53 | 91.53 | 53.13 |
| **E52Skin1** | mRNA | 25414717 | 25108944 | 7.62 | 7.53 | 98.8 | 0.03 | 96.61 | 91.51 | 51.8 |
| **E52Skin3** | mRNA | 30626485 | 30218192 | 9.19 | 9.07 | 98.67 | 0.03 | 96.84 | 92 | 52.75 |
| **E52Skin4** | mRNA | 30901615 | 30688420 | 9.27 | 9.21 | 99.31 | 0.03 | 96.61 | 91.52 | 52.43 |
| **E52Skin10** | mRNA | 28987618 | 28564503 | 8.7 | 8.57 | 98.54 | 0.03 | 96.73 | 91.8 | 52.77 |
| **E52Skin11** | mRNA | 28122788 | 27496126 | 8.44 | 8.25 | 97.77 | 0.03 | 96.77 | 91.97 | 53.55 |
| **E52Skin12** | mRNA | 20797941 | 20612961 | 6.24 | 6.18 | 99.11 | 0.03 | 96.16 | 90.6 | 52.3 |
| **E60Skin1** | mRNA | 29203812 | 28898330 | 8.76 | 8.67 | 98.95 | 0.03 | 96.43 | 91.15 | 52.83 |
| **E60Skin2** | mRNA | 27137294 | 26776370 | 8.14 | 8.03 | 98.67 | 0.03 | 96.69 | 91.71 | 52.58 |
| **E60Skin3** | mRNA | 28305498 | 27927465 | 8.49 | 8.38 | 98.66 | 0.03 | 96.5 | 91.3 | 53.06 |
| **E60Skin5** | mRNA | 27938918 | 27588780 | 8.38 | 8.28 | 98.75 | 0.03 | 96.06 | 90.54 | 54.95 |
| **E60Skin6** | mRNA | 29128972 | 28860261 | 8.74 | 8.66 | 99.08 | 0.03 | 96.53 | 91.43 | 53.48 |
| **E41miRNAskin1** | miRNA | 13887745 | 13887683 | 0.694 | 0.694 | NA | 0.01 | 99.04 | 96.8 | 57.07 |
| **E41miRNAskin2** | miRNA | 13531460 | 13531397 | 0.677 | 0.677 | NA | 0.01 | 99.1 | 96.87 | 53.26 |
| **E41miRNAskin3** | miRNA | 14065373 | 14065292 | 0.703 | 0.703 | NA | 0.01 | 98.68 | 95.87 | 52.61 |
| **E41miRNAskin4** | miRNA | 15808395 | 15808309 | 0.79 | 0.79 | NA | 0.01 | 99.14 | 97.02 | 51.17 |
| **E41miRNAskin5** | miRNA | 14613318 | 14612329 | 0.731 | 0.731 | NA | 0.01 | 99.09 | 96.81 | 51.43 |
| **E41miRNAskin6** | miRNA | 15119668 | 15118630 | 0.756 | 0.756 | NA | 0.01 | 99.13 | 96.9 | 51.46 |
| **E41miRNAheart1** | miRNA | 11835039 | 11834213 | 0.592 | 0.592 | NA | 0.01 | 99.04 | 96.6 | 49.07 |
| **E41miRNAheart2** | miRNA | 12667348 | 12666449 | 0.633 | 0.633 | NA | 0.01 | 99.07 | 96.73 | 48.23 |
| **E41miRNAheart3** | miRNA | 16261395 | 16260327 | 0.813 | 0.813 | NA | 0.01 | 99.03 | 96.61 | 48.57 |
| **E41miRNAheart4** | miRNA | 12636346 | 12635462 | 0.632 | 0.632 | NA | 0.01 | 98.96 | 96.56 | 48.47 |
| **E41miRNAheart5** | miRNA | 11642364 | 11641542 | 0.582 | 0.582 | NA | 0.01 | 99.09 | 96.76 | 50.6 |
| **E41miRNAheart6** | miRNA | 14275111 | 14275056 | 0.714 | 0.714 | NA | 0.01 | 99.09 | 96.77 | 51.15 |
| **E41miRNALiver2** | miRNA | 15501910 | 15501402 | 0.775 | 0.775 | NA | 0.01 | 98.96 | 96.55 | 51.43 |
| **E41miRNALiver3** | miRNA | 13628775 | 13627783 | 0.681 | 0.681 | NA | 0.01 | 99.05 | 96.64 | 49.68 |
| **E41miRNALiver4** | miRNA | 13386743 | 13385823 | 0.669 | 0.669 | NA | 0.01 | 99.05 | 96.64 | 49.62 |
| **E41miRNALiver5** | miRNA | 14758827 | 14757869 | 0.738 | 0.738 | NA | 0.01 | 98.45 | 95.45 | 52.86 |
| **E41miRNALiver6** | miRNA | 14315208 | 14314221 | 0.716 | 0.716 | NA | 0.01 | 99.09 | 96.74 | 51.99 |
| **E41miRNAlung1** | miRNA | 12305303 | 12304482 | 0.615 | 0.615 | NA | 0.01 | 99.01 | 96.65 | 54.23 |
| **E41miRNAlung2** | miRNA | 12693881 | 12693048 | 0.635 | 0.635 | NA | 0.01 | 99.03 | 96.69 | 50.76 |
| **E41miRNAlung3** | miRNA | 14803137 | 14802146 | 0.74 | 0.74 | NA | 0.01 | 99.04 | 96.73 | 48.96 |
| **E41miRNAlung4** | miRNA | 13769943 | 13769045 | 0.688 | 0.688 | NA | 0.01 | 98.84 | 96.35 | 49.2 |
| **E41miRNAlung5** | miRNA | 14584764 | 14583791 | 0.729 | 0.729 | NA | 0.01 | 99.03 | 96.74 | 50.16 |
| **E41miRNAlung6** | miRNA | 13614961 | 13614043 | 0.681 | 0.681 | NA | 0.01 | 99.13 | 96.91 | 49.7 |
| **E41miRNAkidney1** | miRNA | 11680105 | 11679282 | 0.584 | 0.584 | NA | 0.01 | 98.92 | 96.33 | 51.5 |
| **E41miRNAkidney2** | miRNA | 12242876 | 12242012 | 0.612 | 0.612 | NA | 0.01 | 98.95 | 96.34 | 50.78 |
| **E41miRNAkidney3** | miRNA | 15214514 | 15213516 | 0.761 | 0.761 | NA | 0.01 | 99.02 | 96.57 | 51.52 |
| **E41miRNAkidney4** | miRNA | 14204256 | 14203282 | 0.71 | 0.71 | NA | 0.01 | 98.94 | 96.15 | 50.39 |
| **E41miRNAkidney5** | miRNA | 10413838 | 10413097 | 0.521 | 0.521 | NA | 0.01 | 99.05 | 96.66 | 51.39 |
| **E41miRNAkidney6** | miRNA | 10651103 | 10650430 | 0.533 | 0.533 | NA | 0.01 | 98.85 | 96.19 | 52.36 |

Q20: The percentage of bases with Phred value greater than 20 in the total base.

Q30: The percentage of bases with Phred value greater than 30 in the total base.

GC Content: Calculate the percentage of the total number of bases G and C in the total number of bases.

**Supplementary Table2 128 DE mRNAs associated with HFs development and hair placode formation**

| **Gene ID** | **Gene name** | **BaseMean** | **log2FoldChange** | **P value** | **P-adjust** |
| --- | --- | --- | --- | --- | --- |
| ENSSSCG00000007029 | DKK4 | 901.929328 | -1.526916286 | 1.38E-18 | 9.95E-15 |
| ENSSSCG00000037697 | MGP | 709.9325041 | 1.652582061 | 1.90E-15 | 9.16E-12 |
| ENSSSCG00000039408 | ADCY7 | 386.9229724 | -1.19343812 | 1.42E-11 | 1.87E-08 |
| ENSSSCG00000008134 | EDAR | 867.5641253 | -2.112143341 | 3.65E-11 | 3.96E-08 |
| ENSSSCG00000009222 | SPARCL1 | 4697.931095 | 1.522100943 | 3.84E-11 | 3.96E-08 |
| ENSSSCG00000004094 | PPP1R14C | 196.1623082 | -1.641128468 | 4.28E-11 | 4.12E-08 |
| ENSSSCG00000007569 | GRIFIN | 142.9184208 | 1.790882277 | 2.57E-10 | 1.61E-07 |
| ENSSSCG00000038384 | COX4I2 | 2270.71597 | 1.040928171 | 1.22E-09 | 6.09E-07 |
| ENSSSCG00000005250 | APBA1 | 252.3542859 | -1.110546016 | 1.35E-09 | 6.52E-07 |
| ENSSSCG00000010698 | FGFR2 | 1078.526813 | -1.239711452 | 3.15E-09 | 1.26E-06 |
| ENSSSCG00000017204 | ITGB4 | 1567.621136 | -1.281820934 | 1.77E-08 | 5.06E-06 |
| ENSSSCG00000012167 | PHEX | 67.00494594 | 2.747324436 | 6.02E-08 | 1.30E-05 |
| ENSSSCG00000017583 | SGCA | 190.5640001 | 2.178564649 | 6.24E-08 | 1.33E-05 |
| ENSSSCG00000011178 | CPNE4 | 109.4756654 | -1.38759779 | 1.18E-07 | 2.14E-05 |
| ENSSSCG00000009011 | FHDC1 | 528.6024539 | -1.128870592 | 1.19E-07 | 2.14E-05 |
| ENSSSCG00000023289 | GREM2 | 84.09931474 | -1.545563526 | 1.34E-07 | 2.30E-05 |
| ENSSSCG00000038508 | SPTBN2 | 290.7986113 | -1.573950168 | 1.41E-07 | 2.39E-05 |
| ENSSSCG00000035269 | NXNL2 | 348.8534257 | 1.156025851 | 2.67E-07 | 3.86E-05 |
| ENSSSCG00000016018 | FRZB | 354.360002 | 1.480194617 | 3.08E-07 | 4.32E-05 |
| ENSSSCG00000015403 | HGF | 117.0584398 | 1.76709168 | 4.49E-07 | 5.48E-05 |
| ENSSSCG00000007073 | ISM1 | 1385.716328 | -1.057798239 | 1.49E-06 | 0.000119 |
| ENSSSCG00000035093 | KIF26A | 976.7512595 | -1.212571146 | 1.78E-06 | 0.000134 |
| ENSSSCG00000001025 | DSP | 2970.843233 | -1.334095896 | 2.51E-06 | 0.000183 |
| ENSSSCG00000009867 | TBX5 | 191.9142427 | 1.416011647 | 2.56E-06 | 0.000185 |
| ENSSSCG00000012600 | AGTR2 | 113.6636086 | 1.482583708 | 2.71E-06 | 0.000191 |
| ENSSSCG00000031346 | CMKLR1 | 205.9013339 | -1.243279861 | 2.73E-06 | 0.000191 |
| ENSSSCG00000016331 | RAMP1 | 564.9753332 | 1.549248024 | 4.72E-06 | 0.000276 |
| ENSSSCG00000030827 | FGFR3 | 375.8418354 | -1.279165466 | 4.76E-06 | 0.000277 |
| ENSSSCG00000015894 | DPP4 | 158.9644928 | 1.16092404 | 5.06E-06 | 0.000289 |
| ENSSSCG00000016522 | PTN | 18610.25855 | 1.100723456 | 1.42E-05 | 0.000611 |
| ENSSSCG00000024676 | SRPK3 | 278.2455487 | 1.720638467 | 1.60E-05 | 0.000666 |
| ENSSSCG00000017317 | WNT3 | 942.0969481 | -1.065269654 | 1.71E-05 | 0.00069 |
| ENSSSCG00000003768 | NEXN | 1065.958496 | 1.251514457 | 1.96E-05 | 0.000743 |
| ENSSSCG00000003646 | POU3F1 | 119.8289005 | -1.250934841 | 3.60E-05 | 0.001127 |
| ENSSSCG00000007927 | PPL | 400.9345789 | -1.518136571 | 3.95E-05 | 0.001185 |
| ENSSSCG00000027160 | DSG3 | 259.17655 | -1.315477698 | 4.43E-05 | 0.001285 |
| ENSSSCG00000024681 | TECRL | 34.61904012 | 2.932749672 | 5.99E-05 | 0.001614 |
| ENSSSCG00000004236 | PKIB | 56.93580813 | 1.49197361 | 6.16E-05 | 0.001646 |
| ENSSSCG00000009661 | ADRA1A | 143.4325193 | -1.398178297 | 6.74E-05 | 0.001753 |
| ENSSSCG00000024800 | DSC2 | 1015.311881 | -1.124628049 | 7.74E-05 | 0.001923 |
| ENSSSCG00000022739 | DSG2 | 300.9561438 | -1.075679795 | 8.31E-05 | 0.002038 |
| ENSSSCG00000013784 | DNAJB1 | 3817.805115 | 1.017485925 | 8.72E-05 | 0.002116 |
| ENSSSCG00000029621 | BMPR1B | 363.1345683 | -1.163304666 | 9.05E-05 | 0.002159 |
| ENSSSCG00000022490 | GPR83 | 72.39496243 | 1.557257682 | 0.00011 | 0.002437 |
| ENSSSCG00000034973 | CXCL12 | 1429.577786 | 1.066652658 | 0.000129 | 0.002707 |
| ENSSSCG00000035442 | SLC36A1 | 278.802915 | -1.045442242 | 0.00013 | 0.002714 |
| ENSSSCG00000008521 | SRD5A2 | 118.8635786 | 3.802948695 | 0.000175 | 0.003393 |
| ENSSSCG00000024954 | FGF1 | 39.46596482 | 1.52159932 | 0.00019 | 0.003588 |
| ENSSSCG00000036744 | ALX1 | 10.47556165 | 4.514701986 | 0.000204 | NA |
| ENSSSCG00000013313 | PRRG4 | 157.1411685 | -1.241604008 | 0.000246 | 0.004307 |
| ENSSSCG00000004290 | TBX18 | 669.0328034 | 1.040926738 | 0.000253 | 0.004374 |
| ENSSSCG00000015271 | PRELP | 134.1160574 | 1.589490881 | 0.000271 | 0.004625 |
| ENSSSCG00000031027 | IRS4 | 332.5254527 | -1.399445976 | 0.000291 | 0.004881 |
| ENSSSCG00000000602 | RERG | 112.3601573 | 1.367914646 | 0.000468 | 0.006906 |
| ENSSSCG00000020694 | DSG1 | 242.2239009 | -1.157644363 | 0.000474 | 0.006951 |
| ENSSSCG00000028060 | SLC4A8 | 211.995682 | -1.022317647 | 0.00051 | 0.007297 |
| ENSSSCG00000003788 | PTGER3 | 186.1823188 | 1.005305903 | 0.000694 | 0.009183 |
| ENSSSCG00000005287 | PSAT1 | 593.1185365 | 1.096084251 | 0.000699 | 0.009218 |
| ENSSSCG00000021375 | HS6ST2 | 145.2096101 | 1.043317421 | 0.000716 | 0.009391 |
| ENSSSCG00000004614 | UNC13C | 115.5565989 | -1.21646246 | 0.000877 | 0.010818 |
| ENSSSCG00000036724 | CRYAB | 68.83082648 | 1.80357988 | 0.000899 | 0.010965 |
| ENSSSCG00000000281 | HOXC12 | 4.86609837 | -5.567723577 | 0.000917 | NA |
| ENSSSCG00000014047 | FGFR4 | 527.0584504 | 1.933141357 | 0.001099 | 0.012634 |
| ENSSSCG00000003755 | MCOLN2 | 59.79984891 | 1.759435516 | 0.001186 | 0.013337 |
| ENSSSCG00000000709 | PLEKHG6 | 81.20056267 | -1.360667516 | 0.00125 | 0.013865 |
| ENSSSCG00000017904 | ENO3 | 880.0399018 | 1.691179057 | 0.001409 | 0.015032 |
| ENSSSCG00000035524 | WNT9B | 136.9749036 | -1.092879936 | 0.001463 | 0.01543 |
| ENSSSCG00000003539 | GRHL3 | 71.23021238 | -1.29143472 | 0.00152 | 0.015854 |
| ENSSSCG00000017522 | SP6 | 24.5237249 | -2.343235265 | 0.001791 | 0.017644 |
| ENSSSCG00000006187 | MSC | 981.6731975 | 1.64745931 | 0.001885 | 0.018256 |
| ENSSSCG00000017053 | NIPAL4 | 204.9907629 | -1.034156014 | 0.002161 | 0.020003 |
| ENSSSCG00000003744 | MOCOS | 57.61768058 | -1.625259032 | 0.002188 | 0.020149 |
| ENSSSCG00000007485 | BCAS1 | 140.3737914 | 1.371747907 | 0.002254 | 0.020561 |
| ENSSSCG00000003078 | CEACAM19 | 29.99298778 | -1.453133591 | 0.002267 | 0.020606 |
| ENSSSCG00000008745 | PROM1 | 65.39696786 | 1.159063113 | 0.002693 | 0.023095 |
| ENSSSCG00000015550 | RGS16 | 73.46073421 | 1.083262594 | 0.002789 | 0.023611 |
| ENSSSCG00000017112 | IRX4 | 122.5798446 | -1.155227145 | 0.003067 | 0.025253 |
| ENSSSCG00000037269 | LANCL3 | 42.27685716 | 1.236199101 | 0.003088 | 0.025328 |
| ENSSSCG00000027361 | OVOL1 | 34.26175162 | -1.309183853 | 0.00319 | 0.025819 |
| ENSSSCG00000026478 | PADI2 | 49.93806158 | 1.458035913 | 0.003228 | 0.026032 |
| ENSSSCG00000035227 | ESRP2 | 177.8077253 | -1.066544164 | 0.003272 | 0.026336 |
| ENSSSCG00000016286 | PRSS56 | 69.41911651 | 1.361877533 | 0.003486 | 0.02753 |
| ENSSSCG00000022200 | SYT17 | 29.4389876 | 1.378174073 | 0.003602 | 0.028184 |
| ENSSSCG00000031905 | KCNS3 | 98.11648756 | 1.100868725 | 0.003674 | 0.028585 |
| ENSSSCG00000036354 | FOXI3 | 6.344635345 | -3.990274151 | 0.003853 | NA |
| ENSSSCG00000011736 | SLITRK3 | 49.25670871 | -1.073964171 | 0.004249 | 0.031649 |
| ENSSSCG00000000607 | ART4 | 35.74602113 | 2.133279804 | 0.004682 | 0.033959 |
| ENSSSCG00000006746 | VANGL1 | 76.61951928 | -1.049681472 | 0.004725 | 0.034159 |
| ENSSSCG00000008522 | XDH | 11.47829327 | -2.393216177 | 0.004851 | 0.034682 |
| ENSSSCG00000028331 | IL1R2 | 25.1816602 | 1.558840396 | 0.00494 | 0.035137 |
| ENSSSCG00000011717 | IGSF10 | 337.7259674 | -1.088994816 | 0.005574 | 0.038159 |
| ENSSSCG00000000591 | PIK3C2G | 88.16185175 | -1.333381917 | 0.006389 | 0.041792 |
| ENSSSCG00000037395 | CLIC3 | 42.60325182 | -1.649953631 | 0.006521 | 0.042482 |
| ENSSSCG00000016157 | MYL1 | 5680.644743 | 2.391344425 | 0.006877 | 0.044043 |
| ENSSSCG00000000937 | MYF5 | 350.5089032 | 1.880512589 | 0.007016 | 0.044611 |
| ENSSSCG00000030305 | SLC15A2 | 212.277057 | 1.088917785 | 0.007222 | 0.045347 |
| ENSSSCG00000007538 | GATA5 | 49.8489176 | -1.175931729 | 0.008002 | 0.048273 |
| ENSSSCG00000003585 | GJB5 | 27.88220833 | -1.592077743 | 0.008401 | 0.049948 |
| ENSSSCG00000035805 | DLK1 | 25.940653 | 1.738812023 | 0.003994 | 0.030198 |
| ENSSSCG00000014827 | PLEKHB1 | 368.5039787 | 1.880931544 | 4.27E-07 | 5.36E-05 |
| ENSSSCG00000039535 | CAMK2B | 302.7468088 | 1.762003697 | 4.51E-07 | 5.48E-05 |
| ENSSSCG00000005316 | TPM2 | 144.6820936 | -1.31699562 | 9.47E-07 | 9.05E-05 |
| ENSSSCG00000002315 | SLC8A3 | 186.5852773 | -1.327524259 | 4.11E-06 | 0.000255 |
| ENSSSCG00000022833 | FGF16 | 2734.693493 | 4.969690469 | 7.47E-06 | 0.000387 |
| ENSSSCG00000031616 | FOSB | 83.26247621 | 1.956964338 | 1.78E-05 | 0.000711 |
| ENSSSCG00000029061 | BMP3 | 51.05973633 | 1.975889172 | 2.42E-05 | 0.000866 |
| ENSSSCG00000036883 | FABP3 | 28.10569592 | 3.251696656 | 3.39E-05 | 0.001085 |
| ENSSSCG00000034627 | PAX7 | 323.9222115 | -1.093987729 | 4.88E-05 | 0.001383 |
| ENSSSCG00000017256 | ABCA6 | 16.67544704 | 4.792470275 | 5.85E-05 | 0.001583 |
| ENSSSCG00000014876 | MYO7A | 78.84860217 | -1.287368494 | 7.82E-05 | 0.001939 |
| ENSSSCG00000026068 | GRHL1 | 80.78673687 | -1.485726527 | 8.31E-05 | 0.002038 |
| ENSSSCG00000010447 | ACTA2 | 932.4027066 | -1.087306034 | 9.77E-05 | 0.00227 |
| ENSSSCG00000007799 | MYLPF | 56.34057872 | 2.663110913 | 0.000221 | 0.003964 |
| ENSSSCG00000016720 | PGAM2 | 126.117844 | 1.029986721 | 0.000329 | 0.005354 |
| ENSSSCG00000040636 | KRT80 | 47.5897181 | -1.537844993 | 0.000507 | 0.007276 |
| ENSSSCG00000002007 | FITM1 | 125.7475953 | -1.260150358 | 0.000513 | 0.007332 |
| ENSSSCG00000000371 | PMEL | 151.0149028 | 3.77013421 | 0.000687 | 0.009097 |
| ENSSSCG00000038152 | CNTN6 | 271.9313803 | 1.148277248 | 0.000708 | 0.009311 |
| ENSSSCG00000004093 | IYD | 29.17831291 | 2.325850196 | 0.000711 | 0.00934 |
| ENSSSCG00000001485 | MLIP | 701.0351939 | 3.926525877 | 0.000977 | 0.011626 |
| ENSSSCG00000008524 | GALNT14 | 58.25550648 | -1.221594741 | 0.000996 | 0.011802 |
| ENSSSCG00000033260 | TNNI2 | 18.76839069 | 3.726022899 | 0.001308 | 0.014256 |
| ENSSSCG00000014418 | STK32A | 333.1905626 | -1.046988633 | 0.001321 | 0.01438 |
| ENSSSCG00000006489 | TMEM79 | 43.41219574 | -1.397336343 | 0.001344 | 0.014558 |
| ENSSSCG00000009494 | SOX21 | 45.28876791 | 1.193470654 | 0.002014 | 0.019033 |
| ENSSSCG00000035971 | DUSP2 | 19.35168038 | 1.75338578 | 0.002484 | 0.021846 |
| ENSSSCG00000036893 | PTHLH | 106.2937197 | -1.172241746 | 0.002487 | 0.021858 |
| ENSSSCG00000022891 | MYMK | 57.69260167 | 1.069137331 | 0.003236 | 0.026079 |

**Supplementary Table 3 Primers used for the quantitative real-time PCR analysis**

| **Primer** | **Sequence (5’→3’)** |
| --- | --- |
| **ssc-miR-146b** | GCAGTGAGAACTGAATTCCA |
|  | TCCAGTTTTTTTTTTTTTTTGCCT |
| **ssc-miR-29a-5p** | GCAGACTGATTTCTTTTGGTGT |
|  | GGTCCAGTTTTTTTTTTTTTTTCTGA |
| **ssc-miR-186-5p** | CGCAGCAAAGAATTCTCCT |
|  | GGTCCAGTTTTTTTTTTTTTTTAAGC |
| **ssc-miR-9785-5p** | ACCCTCATCCTGGTGTC |
|  | GTCCAGTTTTTTTTTTTTTTTGGTG |
| **ssc-miR-10387** | GCTGATAGAGCTCCTGGA |
|  | CCAGTTTTTTTTTTTTTTTAGGCCTT |
| **ssc-miR-10386** | GGTCGTCCTCTCCCTC |
|  | AGGTCCAGTTTTTTTTTTTTTTTAGG |
| **ssc-miR-150** | CTCCCAACCCTTGTACCA |
|  | GGTCCAGTTTTTTTTTTTTTTTCACT |
| **ssc-miR-122-5p** | GTGGAGTGTGACAATGGTG |
|  | AGGTCCAGTTTTTTTTTTTTTTTACAA |
| **ssc-miR-10387** | GCTGATAGAGCTCCTGGA |
|  | CCAGTTTTTTTTTTTTTTTAGGCCTT |
| **ssc-miR-129a-5p** | CAGCTTTTTGCGGTCTG |
|  | TCCAGTTTTTTTTTTTTTTTGCAAG |
| **NPTX1** | CTGGAGAACCTCGAGCAGTATAG |
|  | GCGGTTGTCCTTCTGACCTT |
| **IGFBP1** | ACATCAAGAAGTGGAAGGAGCC |
|  | CCGTTCTTGTTGCAGTTCGG |
| **EDAR** | CCCTGTCTCCCTGGCTACTA |
|  | GAAGTTGGCTGACACTCCCA |
| **HOXC12** | GCTACGCAAAAGGGTCTCCT |
|  | TGAACCTGAGCATCGTCCAC |
| **CHGA** | CACTCCGAGGAGATGAACGG |
|  | ACTCAGTCTGGTCGTTCTGC |
| **STMN1** | GAGCTGATTCTCAGCCCTCG |
|  | ACCTCAGCTTCATGGGACTTG |
| **SERPINA1** | CCGGGCAGTGAAGGACAAT |
|  | GTACAGGCTGAAGGCGAAGT |
| **EHF** | GCTTCCCCTCCATTAGCCAC |
|  | CCCGCTGGAAACATTGCAC |
| **DCHS2** | ATTGATGCTGCGAATGGCAC |
|  | CGTGTGTGTTTTCACGGGTC |
| **FOXQ1** | TGACTCCACCACAGGGGTTA |
|  | AACGGAGAAAGCGTACCTGG |
| **BMPR1b** | AGAGTGGTCAAGAGAGTGAACG |
|  | TGTTATCAAGGAAGTTTGCTTCTGG |
| **EDA** | AGCCATCCAAGTCAAGAATGATCT |
|  | GGCGAAGTCAGTGAAGTTGATATAG |
| **NF-ĸB** | GGCTACCCTGGCACAGAAAT |
|  | GCCTGAGAGGTGGTCTTCAC |
| **Wnt10b** | TCACGCCTACTCTGTCCTCT |
|  | GCTGGAAGGTGCAGGCAT |
| **BMP4** | CGCAGGGACCTATGGAGC |
|  | GGAGGGACACAAGGCGAG |
| **EDARADD** | TCTCCGGACGACCCTCTG |
|  | CCCTGGTGCTGCATATCTGA |
| **LHX2** | CAATGAGAACGACGCGGAAC |
|  | GGCGTTCTGAAACCAGACCT |
| **FGF20** | CCACAGCCTCTTCGGTATCC |
|  | GGCCAGTGTCTCCATGCTTA |
